# Novel benzofuran derivative DK-1014 attenuates lung inflammation via blocking of MAPK/AP-1 and AKT/mTOR signaling in vitro and in vivo

**DOI:** 10.1038/s41598-018-36925-9

**Published:** 2019-01-29

**Authors:** Xuezhen Xu, Ok-Kyoung Kwon, In-Sik Shin, Jyotirling R. Mali, Dipesh S. Harmalkar, Yourim Lim, Gilhye Lee, Qili Lu, Sei-Ryang Oh, Kyung-Seop Ahn, Hye-Gwang Jeong, Kyeong Lee

**Affiliations:** 1 College of Pharmacy, Dongguk University-Seoul, Goyang, 10326 Korea; 2 Department of Toxicology, College of Pharmacy, Chungnam National University, Daejeon, 34134 Korea; 3 Natural Medicine Research Center, Korea Research Institute of Bioscience and Biotechnology, Chungju-si, Chungbuk 28116 Korea; 4 College of Veterinary Medicine, Chonnam National University, Gwangju, 61186 Korea

## Abstract

Benzofuran derivatives have wide range of biological activities as anti-oxidant, anti-inflammatory and anticonvulsant agent. In this study, we investigated whether the novel benzofuran derivative, DK-1014 has the anti-inflammatory effects on macrophage and lung epithelial cells and anti-asthmatic effects on ovalbumin-treated mice. A series of 2-arylbenzofuran analogues were synthesized and evaluated for NO and interleukin-6 (IL-6) inhibition in LPS-stimulated Raw264.7 cells. Of these analogues, compounds 8, 22a, 22d, and 22 f (DK-1014) exhibited notable inhibitory activity with respect to IL-6 and NO production. In particular, compound DK-1014 strongly reduced IL-6, IL-8, and MMP-9 mRNA expression and IL-6, IL-8, and MCP-1 production in phorbol myristate acetate stimulated A549 cells, reduced MAPKs phosphorylation and c-fos translocation, and attenuated AKT, p70S6K and GSK phosphorylation. *In vivo* experiments were also performed on ovalbumin-sensitized and challenged BALB/c mice. DK-1014 reduced the airway hyperresponsiveness, inflammatory cell counts and cytokine levels (IL-4, 5, 13) in bronchial alveolar lavage fluid (BALF) and immunoglobulin E in serum, and attenuated inflammatory cell infiltration and mucus hypersecretion in lung tissue. These findings indicate that **DK-1014** can protect against allergic airway inflammation through the AP-1 and AKT/mTOR pathways and could be useful source for the development of a therapeutic agent for asthma.

## Introduction

Asthma is a serious health concern that affects up to 18% of the population worldwide^1^. It is a common and long-term inflammatory disorder of the airway and lung that often causes inflammation and excessive mucus build up in response to allergens or other triggers. Pulmonary inflammation is characterized by elevated serum IgE and cytokine levels, airway hyper-responsiveness (AHR), airway eosinophilia, and mucus accumulation^2,3^. These inflammatory mediators, including proinflammatory cytokines and monocyte chemoattractant protein-1 (MCP-1), are produced by macrophages and airway epithelial cells via a series of inducible genes, leading to the infiltration of inflammatory cells into airway inflammation^4^. In addition, inflammatory cells produce nitric oxide (NO) or inflammatory cytokines and chemokines, which can be useful markers for airway inflammation^5,6^. Thus, the models for treatment of murine macrophage (Raw 264.7 cells) and human lung epithelial cells (A549 cells) have been widely used to evaluate anti-inflammatory agents for asthma and to elucidate their mechanism^7–10^.

Recent studies have proposed MMPs (matrix metalloproteinase) as important inflammation regulators^11^. It has been reported that their expression can be stimulated by various agents, such as growth factors, inflammatory cytokines, and phorbol myristate acetate (PMA) PMA-induced MMP-9 overexpression is modulated by transcription factor activation such as activator protein-1 (AP-1) and NF-kB through MAPKs (e.g., ERK, JNK and p38) and phosphoinositide 3-kinase (PI3K)/AKT signaling pathways^12–14^. Eventually, MMPs damages the normal alveolar structure, leading to emphysema. Therefore, agents that can suppress AP-1 and MMP-9 activity could be useful for asthma treatment^15^.

It has been reported that the PI3K-AKT-mTOR signaling pathway is mutated or hyper-activated in human cancer. Since the effect of PI3K on intracellular calcium activates cell migration, PI3Kgamma is also believed to be the primary target for chemokine-induced neutrophil movement and chemotactic eosinophilia in several lung disorders, including chronic obstructive pulmonary disorder (COPD)^16^ and asthma^17^. PI3K activates the AKT serine/threonine kinase pathway via direct TSC2 phosphorylation. RSK kinase also phosphorylates mTORC1, Raptor, and p70S6 kinase subunits as well as eEF2k and its downstream effectors. RAS-RAF-MKK1/2-Erk1/2 pathway activation triggers subsequent TSC2 phosphorylation. p38 also activates mTORC- and ERK-mediated TSC2 phosphorylation^18^. Recent studies have found that several pan PI3K inhibitors, including LY294002 and mTOR inhibitor rapamycin, attenuate allergic airway inflammation by suppressing mTOR and p70S6K phosphorylation in mice that have inhaled ovalbumin (OVA)^19^.

Benzofuran heterocycles are key components found in a variety of natural products, particularly in the *Styrax* family, belonging to the class of dibenzylbutane derivatives called neolignans or norneolignans^20^. Some benzofurans have been investigated closely and found to display anticancer, antimicrobial, immune modulatory, antioxidant, and anti-inflammatory activity^21^. The 2-arylbenzofuran scaffold is commonly found in naturally occurring egonols, including homoegonol. Homoegonol isolated from *Styrax japonica*, a medicinal plant widely used for the treatment of inflammatory diseases in Korea. Reportedly, homoegonol exhibits an inhibitory effect on cyclooxygenase-1 and 2 and is also known as a proinflammatory mediator^22^. Our earlier studies have shown that homoegonol effectively suppresses asthmatic responses induced by OVA challenge^23^.

Considering the multifarious potential applications of homoegonol for development of anti-inflammatory agent, we report in this study synthesis and structure-activity relationship of a series of homoegonol analogues with 2-arylbenzofuran scaffolds and *in vitro* assays of IL-6 and NO production inhibitory activity in Raw264.7 cells. But the novel 2-arylbenzofuran analogue DK-1014 is demonstrated to have protective effects against the inflammation of human lung epithelial cells and in asthmatic mice, making it a particularly interesting candidate for the treatment of inflammatory disease.

## Results and Discussion

### Chemistry

Many approaches to the synthesis of homoegonol have been reported^24–28^, with most based on the construction of a benzofuran backbone using the Sonogashira coupling of *O*-halophenols with a palladium catalyst, e.g. PdCl_2_(PPh_3_)_2_^24^. Other synthetic routes include cross-McMurry coupling of a substituted salicylaldehyde with an aromatic aldehyde using low-valent titanium, e.g. TiCl_4_/Zn and TiCl_4_/Mn followed by the oxidative cyclization of *O*-vinylphenols^25,26^; domino cyclization of dibromo vinylphenol with triarylbismuth reagents using Pd(PPh_3_)_4_ in the presence of Cs_2_CO_3_^27^ and cross pinacol-type coupling of a substituted salicylaldehyde with aryl aldehyde using TiCl_4_/Mn followed by acid-promoted diol cyclization^28^. However, these methods have a number of drawbacks, including expensive reagents, tedious workup, and metallic waste.

Our synthetic strategy was modified from that used by Chen *et al*.^24^, which involves the Sonogashira coupling reaction of *o*-iodophenol with alkynes in the presence of Pd/C as a catalyst. Utilizing this process, a series of 2-arylbenzofurans, including homoegonol (**8**) and the analogues **12**–**15**, **17a-b**, and **22a-q**, were readily synthesized. (Supplemently Information).

### Benzofuran derivative effects on LPS-induced inflammation in Raw264.7 cells

Homoegonol **8** and its newly synthesized 2-arylbenzofurans (**12**–**15**, **17a-b**, and **22a-q**) were evaluated for their inhibitory effects on NO and IL-6 production in LPS-stimulated Raw264.7 cells. The Raw264.7 cells were first co-treated with or without the compounds at a concentration of 40 μM for 1 h and LPS (0.5 μg/ml) for 24 h, after which the NO concentration in the supernatant was determined using Griess assays, as summarized in Table 1. Cell viability measured using MTT assays demonstrated that some compounds had no significant cytotoxicity at concentrations where they effectively inhibited NO and IL-6 production.

**Table 1:** Inhibitory activity of benzofuran derivatives in LPS-induced inflammation^a^.

	
Code No.	Structure		at 40 µM		IL-6 inhibition (IC_50_, µM)
	R_1_	R_2_	NO inhibition (%)	Viability (%)	
**8**			13.26 ± 0.84	43.69 ± 0.49	19.94 ± 1.26
**10**	—		16.59 ± 3.83	56.55 ± 3.50	N.D.
**12**			23.08 ± 5.97	76.92 ± 4.85	48.69 ± 4.55
**13**			33.84 ± 1.83	62.34 ± 1.60	28.30 ± 0.07
**14**			33.53 ± 2.50	95.45 ± 15.18	35.61 ± 0.12
**15**			30.22 ± 0.27	7.21 ± 0.01	>40
**17a**			−3.40 ± 6.03	36.19 ± 2.20	>40
**17b**			−1.05 ± 0.17	25.71 ± 0.69	>40
**22a**			12.94 ± 2.29	95.28 ± 2.25	15.89 ± 0.06
**22b**			41.88 ± 2.67	92.76 ± 8.04	>40
**22c**			24.58 ± 2.96	105.93 ± 0.55	>40
**22d**			17.99 ± 0.65	103.31 ± 3.41	20.31 ± 0.01
**22e**			12.86 ± 3.53	94.80 ± 1.20	>40
**22 f (DK-1014)**			30.36 ± 9.82	101.30 ± 1.48	16.19 ± 0.20
**22 g**			93.32 ± 1.88	8.34 ± 3.42	8.34 ± 3.42
**22 h**			68.52 ± 2.14	8.44 ± 1.44	>40
**22i**			78.37 ± 1.01	8.48 ± 0.69	>40
**22j**			18.91 ± 6.94	98.93 ± 14.41	>40
**22k**			25.25 ± 8.16	95.99 ± 6.08	>40
**22 l**			86.23 ± 0.45	0.49 ± 0.02	24.19 ± 6.48
**22 m**			21.92 ± 2.35	114.97 ± 1.09	>40
**22n**			36.51 ± 5.44	55.62 ± 2.36	>40
**22o**			17.58 ± 0.50	77.04 ± 12.72	>40
**22p**			53.83 ± 2.70	5.21 ± 3.61	>40
**22q**			90.12 ± 2.03	10.82 ± 5.2	>40

^a^Values are the mean of three experiments.

N.D., not determined.

Our optimization strategy for the 2-arylbenzofuran analogues was to first modify the hydroxyl alkyl side chain (R_1_), and aryl substitution (R_2_), as shown in Table 1, and then combine the best substituents into a single compound with an improved overall profile. The detailed SAR study have shown in the supporting information.

Among the 2-arylbenzofuran derivatives, **22 f** (referred to as **DK-1014**) was selected as the representative compound for subsequent testing because it demonstrated the potent inhibition of IL-6 and NO production with little effect on cell viability at 40 μM.

### DK-1014 effects on PMA-induced expression and production of cytokines

**Figure 1:**
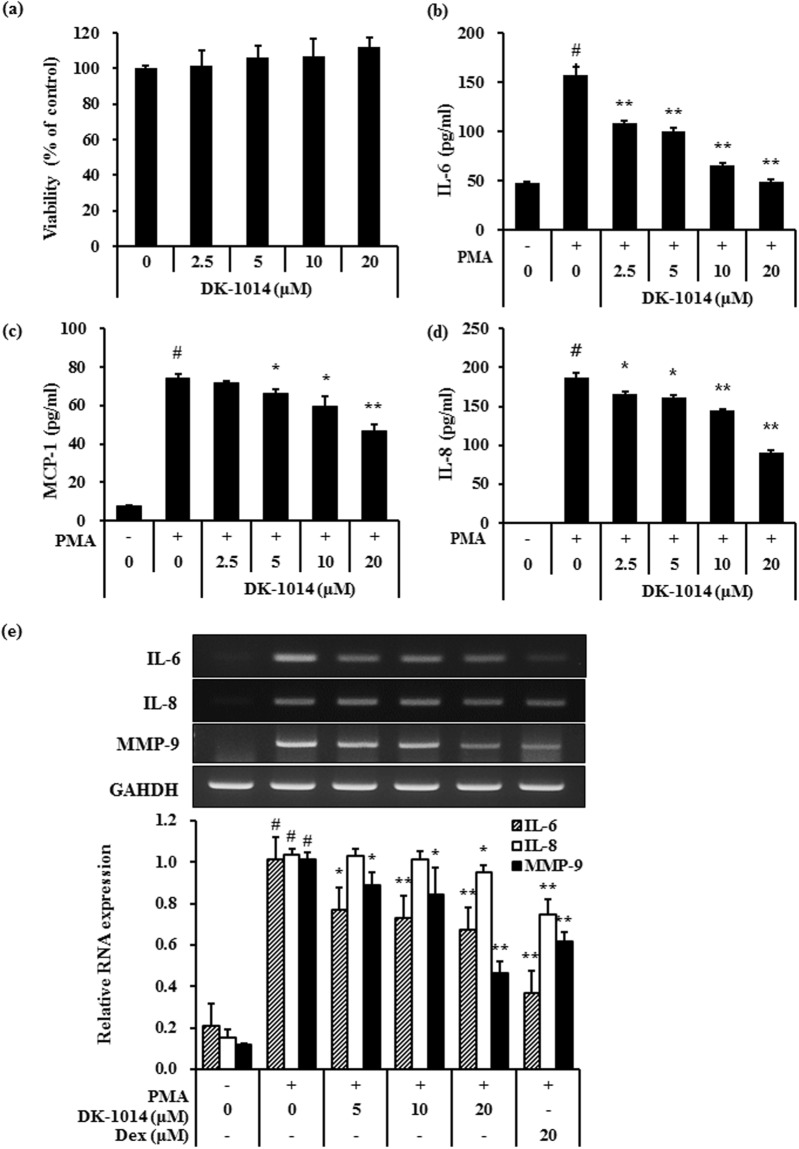
Cell viability and cytokine expression in A549 cells treated with DK-1014. (**a**) A549 cells were incubated with DK-1014 for 24 h and cell viability was detected using MTT; the A549 cells were pre-treated with DK-1014 for 1 h and then incubated for 12 h with PMA (5 nM). Vehicle control cells was treated with 0.1% DMSO in PBS. (**b**–**d**) Cytokines released into the supernatant quantified using ELISA. (**e**) The mRNA levels of IL-6, 8, and MMP-9 quantified using RT-PCR. The data is expressed as the mean ± SD from three independent experiments. ^#^p < 0.05 compared to DMSO-treated cells; *p < 0.05 and **p < 0.005 compared to PMA-treated cells.

Cell viability at different **DK-1014** concentrations was investigated to confirm the appropriate concentration prior to verifying its anti-inflammatory effects in A549 cells. **DK-1014** did not affect cell viability up to 20 μM (Fig. 1a) and then decreased IL-6, IL-8, and MCP-1 production in a dose-dependent manner, all of which were significantly higher in the PMA-treated cell supernatant (Fig. 1b–d). Because **DK-1014** inhibited cytokine production, we then investigated cytokine gene expression. mRNA expression of IL-6, IL-8, and MMP-9 only increased with PMA treatment but was significantly inhibited by **DK-1014** pretreatment (Fig. 1e).

### DK-1014 effects on PMA-induced MAPK and AP-1 activation

**Figure 2:**
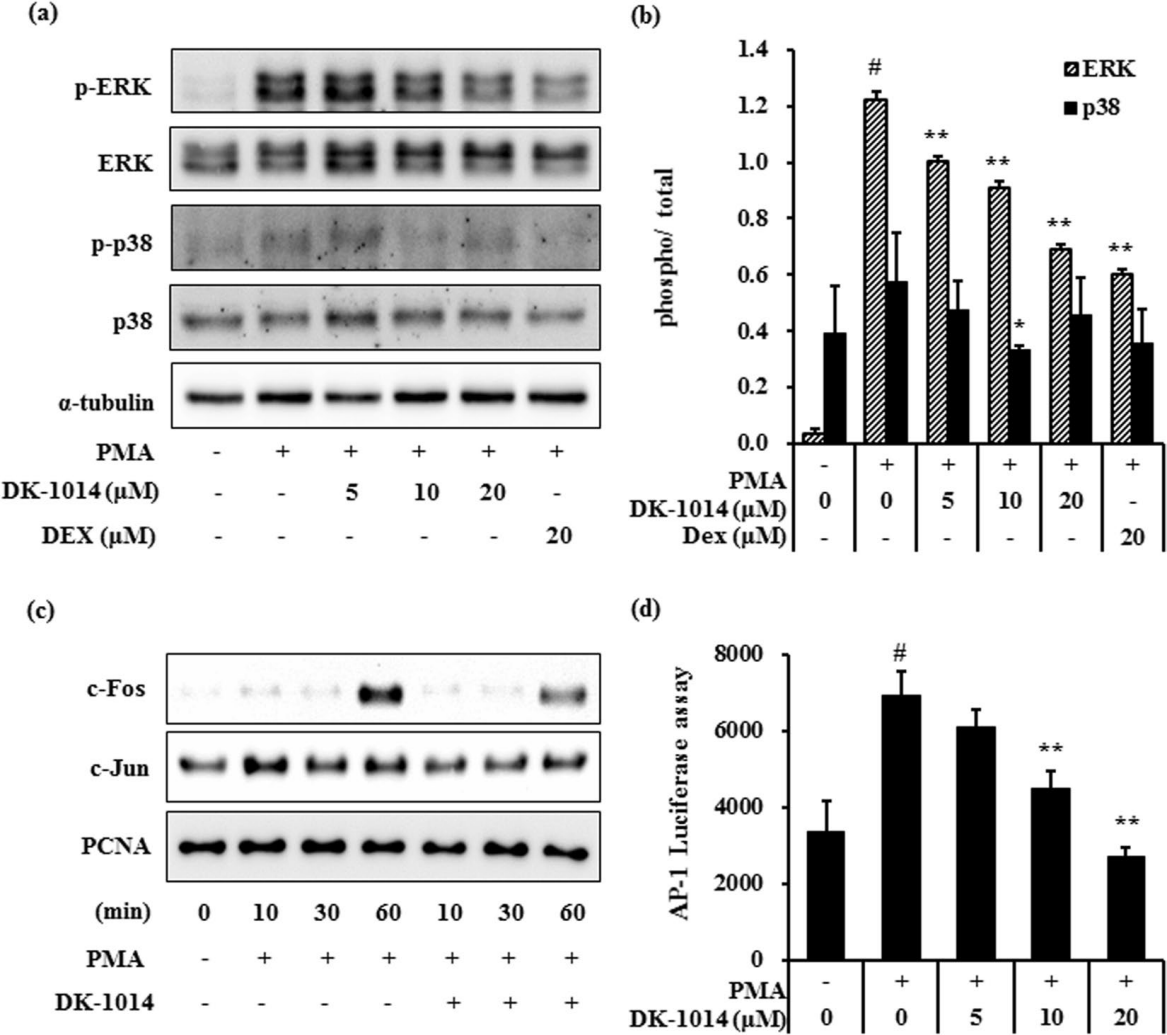
Phosphorylation of MAPK and AP-1 activation in A549 cells treated with DK-1014. A549 cells were pretreated with **DK-1014** or Dexamethasone (DEX) for 1 h and then incubated for 30 min with PMA (5 nM). (**a**) Phosphorylation of MAPKs was evaluated using western blot analysis and (**b**) quantified using ImageJ. (**c**) Nuclear extracts were isolated from the cells using a Pierce kit. (**d**) AP-1 promoter activity detected using luciferase assays. The data is expressed as the mean ± SD from three independent experiments. ^#^p < 0.05 compared to DMSO-treated cells; **p < 0.005 compared to PMA-treated cells.

MAP kinase has been implicated in inflammatory mediator production in various cells. ERK activation induces NF-kB activation and crosstalk between the ERK and AP-1 pathways^29^. AP-1 is major transcription factor that regulates the expression of pro-inflammatory cytokine^30^, thus we investigated whether **DK-1014** affected on AP-1 and MAP kinase signaling pathway for IL-6 production using western blot analysis. **DK-1014** significantly suppressed PMA-induced ERK and p38 kinase phosphorylation as dexamethasone (DEX) (Fig. 2a,b). Additionally, DK-1014 attenuated the translocation of c-fos and c-jun to the nucleus and the AP-1 luciferase activity by PMA (Fig. 2c,d). Thus, the inhibition of IL-6 expression by **DK-1014** is the result of not only ERK and p38 phosphorylation but also AP-1 activation blockade in PMA-treated A549 cells.

### DK-1014 effects on PMA-induced AKT and mTOR pathway phosphorylation

**Figure 3:**
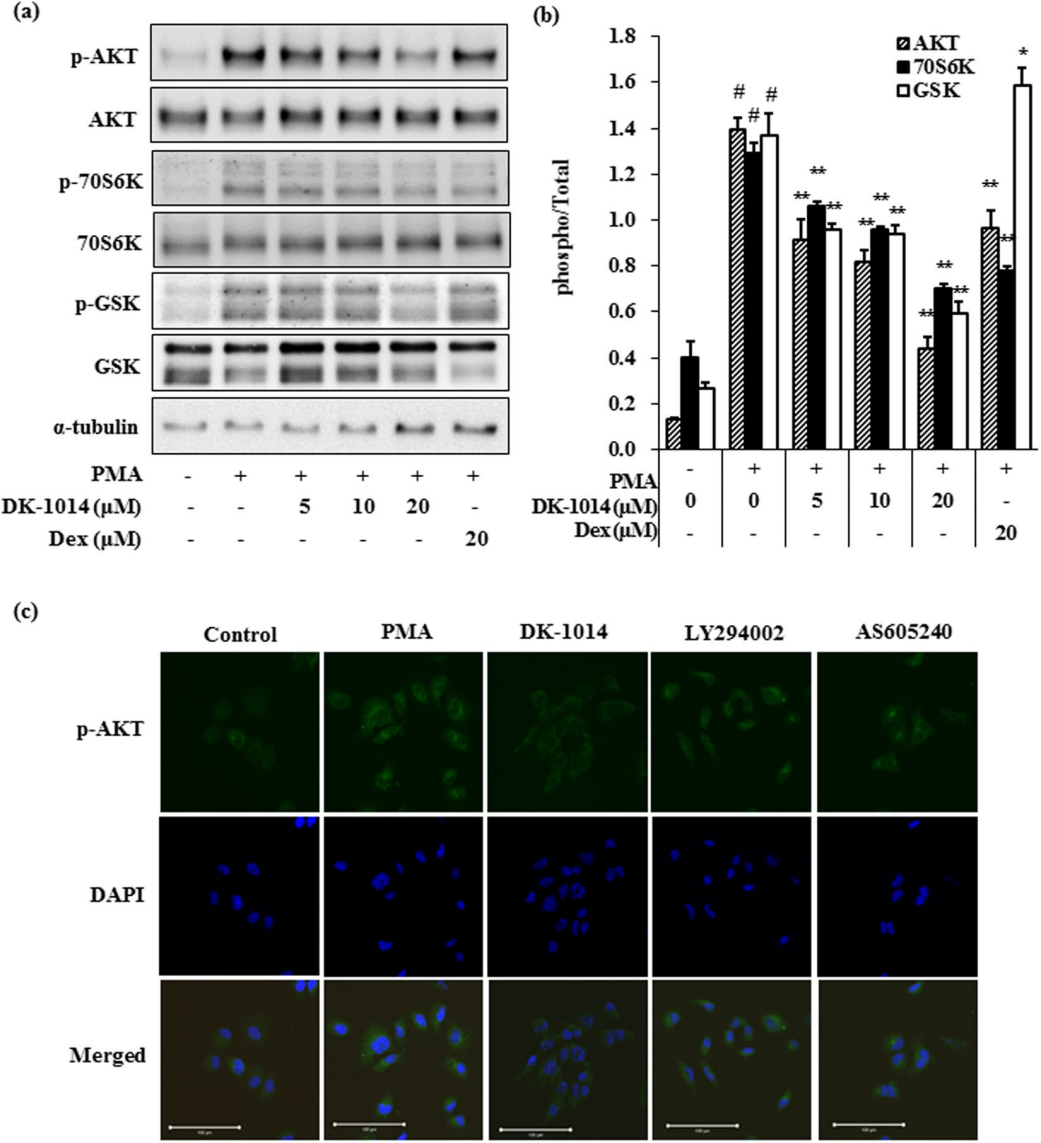
Phosphorylation of AKT/p70S6K in A549 cells treated with **DK-1014**. A549 cells were pre-treated with **DK-1014** or DEX for 1 h and then incubated for 30 min with PMA (5 nM). (**a**) Phosphorylation of AKT/mTOR protein evaluated using western blot analysis and (**b**) quantified using ImageJ. (**c**) Immunocytochemical staining with anti-phosphoAKT antibodies for A549 cells that were **DK-1014** treated or inhibitors (magnification = ×100, bar; 100 μm). ^#^p < 0.05 compared to DMSO-treated cells; **p < 0.005 compared to PMA-treated cells.

AKT overexpression is associated with inflammation^31^ and the mTOR pathway is important in allergic asthma^32^. Therefore, we used immunoblot assays and immunocytochemistry to establish whether **DK-1014** suppresses the AKT/mTOR signaling pathway. Figure 3a shows that **DK-1014** inhibited AKT phosphorylation and mTOR (GSK and 70S6K) downstream components in PMA-treated A549 cells in a dose-dependent manner. Immunocytochemistry analysis found that levels of phosphor-AKT were lower in **DK-1014**-pretreated cells than in PI3K inhibitors (LY294002 and AS605240) treated cells, compared to PMA-treated A549 cells.

### DK-1014 effects on airway hyperresponsiveness in OVA-sensitized/challenged mice

**Figure 4:**
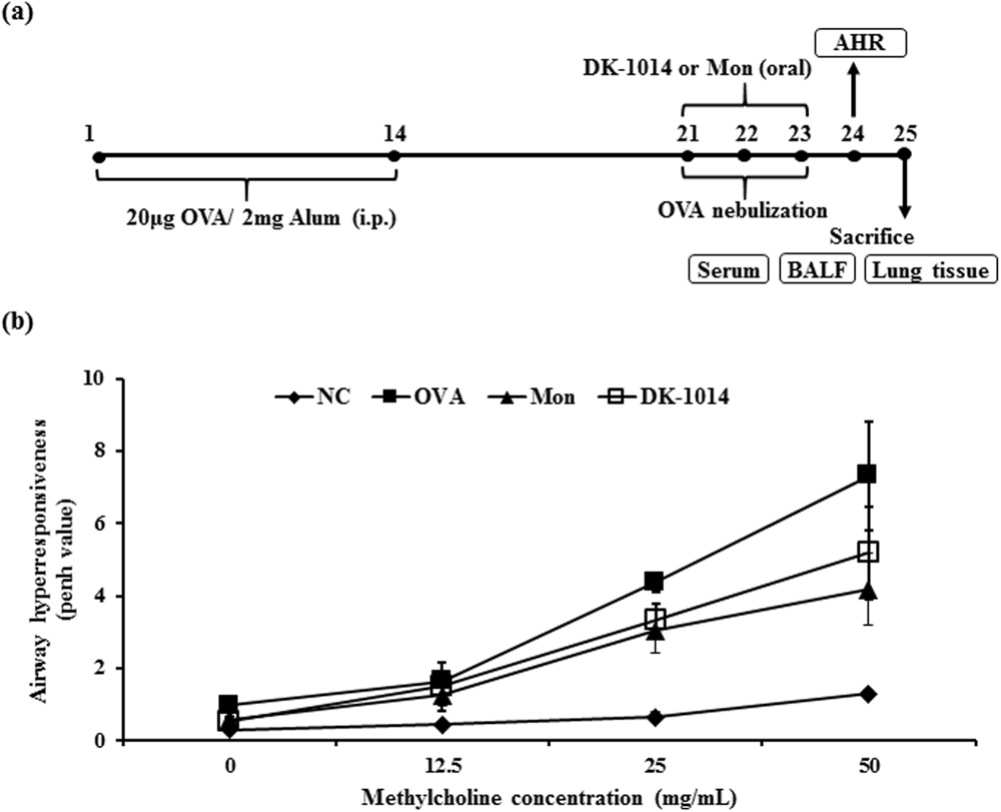
Airway inflammatory mice model and airway hyperresponsiveness in mice exposed to ovalbumin and **DK-1014**. Mice were sensitized with a 20-µg intraperitoneal injection of OVA in aluminium hydroxide in PBS on days 1 and 14: (**a**) airway challenge performed using an ultrasonic nebulizer that administered 30 mg/kg/day of **DK-1014** or Montelukast on days 21, 22, and 23. (**b**) AHR indirectly assessed 24 h after the last challenge using single-chamber, whole-body plethysmography. NC = normal control mice treated with PBS only; OVA = ovalbumin-sensitized/challenged mice; Mon = ovalbumin sensitized/challenged mice treated with montelukast (30 mg/kg); **DK-1014** = ovalbumin sensitized/challenged mice treated with **DK-1014** (30 mg/kg). ^##^p < 0.005 compared with the normal control; *p < 0.05 and **p < 0.005 compared to OVA mice.

To investigate the effects of **DK-1014** on OVA-induced asthmatic mice, the mice were sensitized and neubilized with OVA and orally administered DK-1014 by scheme (Fig. 4a). In asthmatic (OVA) mice, AHR was markedly elevated with an increase in methylcholine concentrations compared with the controls. However, montelukast-treated (Mon) mice exhibited significantly reduced AHR compared with OVA mice for methylcholine concentrations of 25 and 50 mg/ml. Similarly, **DK-1014**-treated mice exhibited significantly lower AHR compared with OVA mice at these same concentrations (Fig. 4b).

### DK-1014 effects on the number of inflammatory cells and Th2 cytokine production in BALF

**Figure 5:**
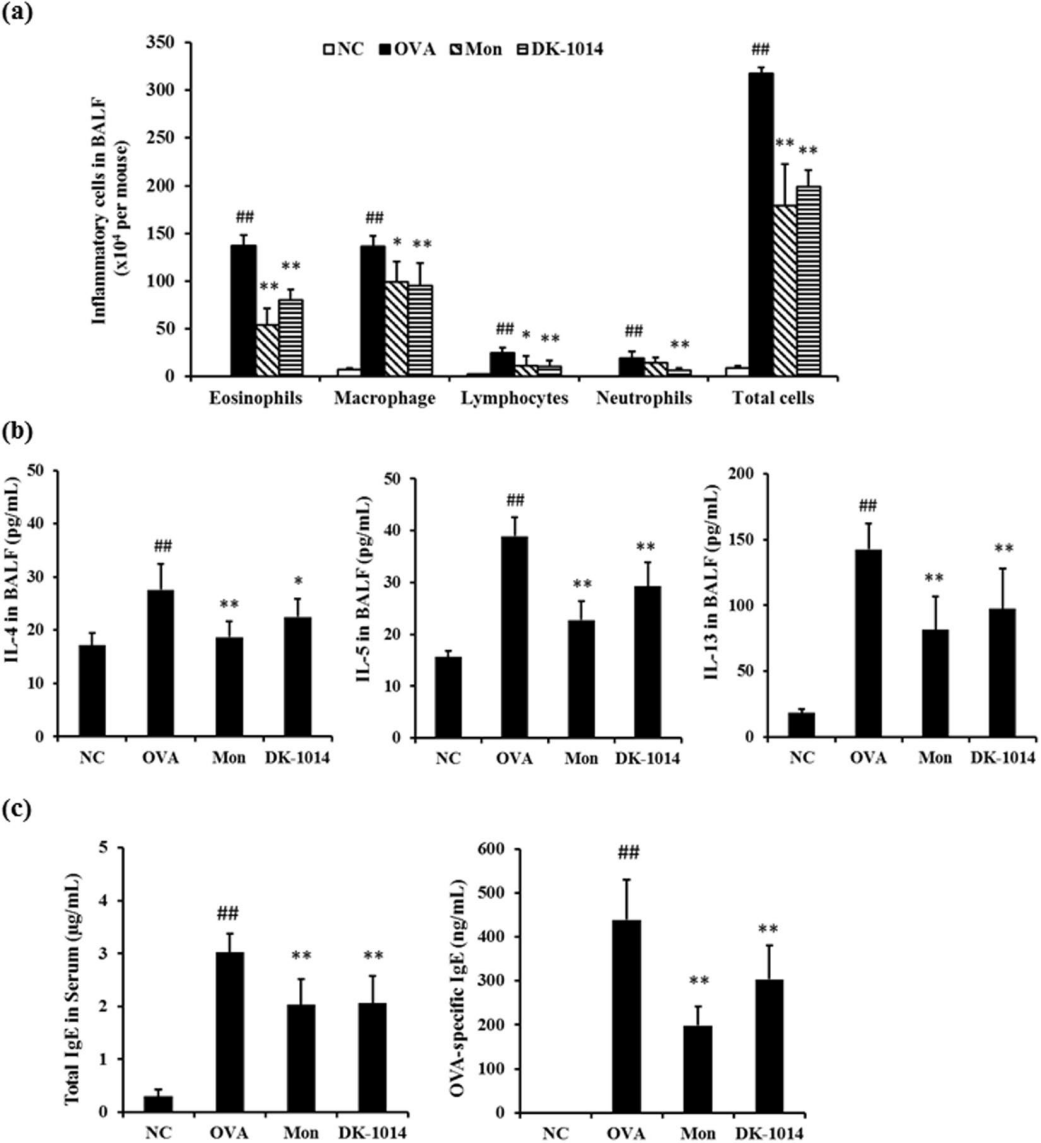
Inflammatory cells, cytokines, and IgE in sensitized mice exposed to ovalbumin and **DK-1014**. Inflammatory cells were collected using centrifugation and stained with Diff-Quik stain reagent. (**a**) Cell count was determined by counting the cells in at least five hemocytometer squares using a light microscope after excluding dead cells using Trypan blue. (**b**) Cytokines in BALF quantified using an ELISA kit. (**c**) Total IgE and OVA-specific IgE in serum quantified using an ELISA kit. NC = normal control mice treated with PBS only; OVA = ovalbumin sensitized/challenged mice; Mon = ovalbumin sensitized/challenged mice treated with montelukast (30 mg/kg); **DK-1014** = ovalbumin sensitized/challenged mice treated with **DK-1014** (30 mg/kg). ^##^p < 0.005 compared to the normal control; *p < 0.05 and **p < 0.005 compared to OVA mice.

Asthmatic mice exhibited a marked increase in inflammatory cell count, particularly for eosinophils, in bronchial alveolar lavage fluid (BALF) compared to normal mice (Fig. 5a), whereas Mon mice had a significantly lower inflammatory cell count compared with OVA mice. Compound **DK-1014**-treated mice also exhibited a significant reduction in inflammatory cell count compared to OVA mice.

Levels of Th2 cytokines (IL-4, 5 and 13) markedly increased in OVA mice compared to normal mice. However, Mon mice exhibited markedly lower Th2 cytokine levels compared with OVA mice. Compound **DK-1014**-treated mice also had a significant reduction in cytokine levels compared to OVA mice (Fig. 5b).

### DK-1014 effects on total IgE and OVA-specific IgE in serum

Total IgE and OVA-specific IgE in serum significantly increased in OVA mice (Fig. 5c). In contrast, Mon and **DK-1014**-treated mice exhibited significantly reduced total IgE and OVA-specific IgE production.

### DK-1014 effects on airway inflammation and mucus production in lung tissue

**Figure 6:**
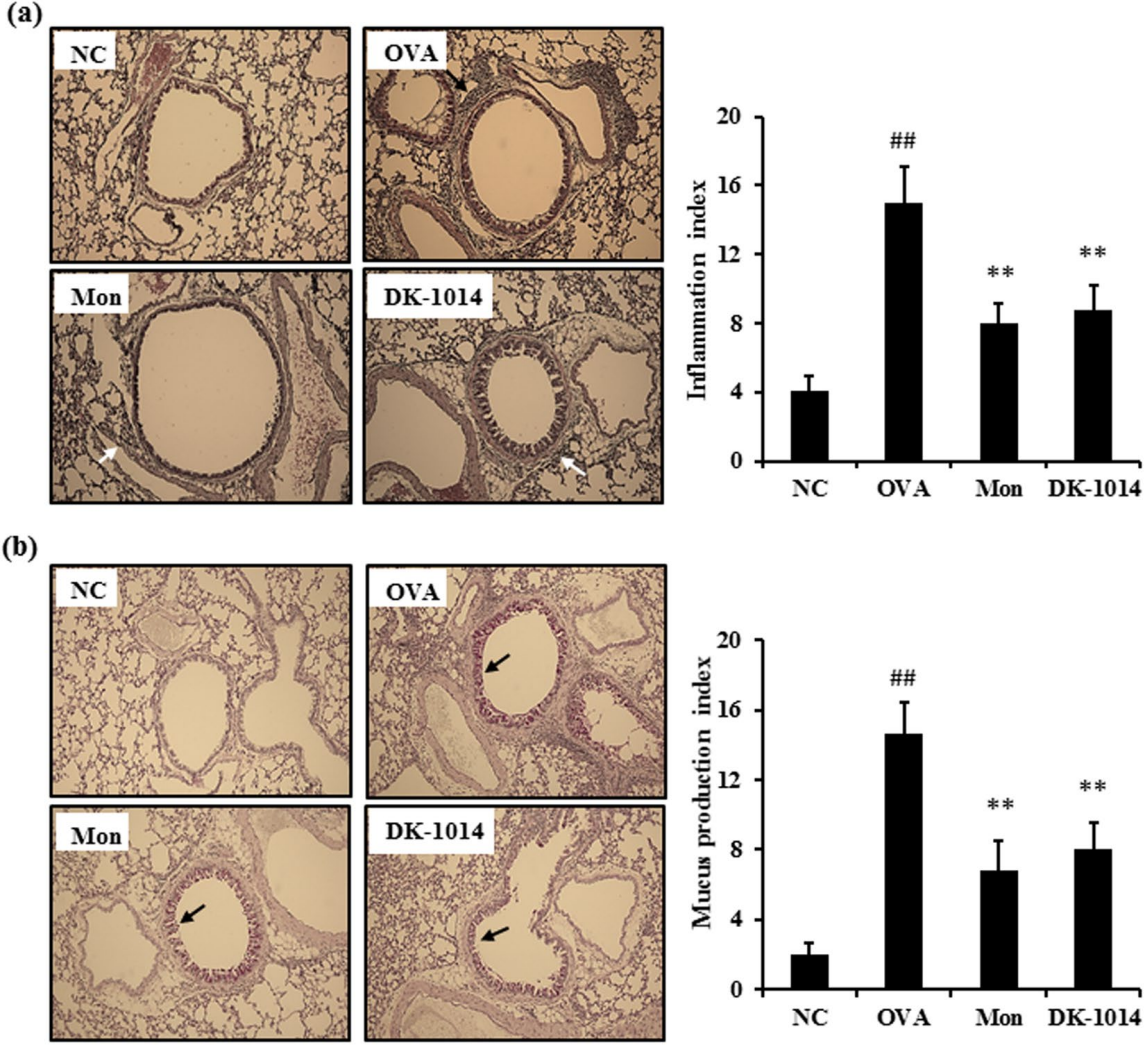
Lung tissue in sensitized mice exposed to ovalbumin and **DK-1014**. (**a**) Histological examination of lung tissue performed using H&E staining for airway inflammation (arrows indicate inflammatory cells). (**b**) PAS staining for mucus secretion (arrows indicate mucus cells). Original magnification was ×100. NC = normal control mice treated with PBS only; OVA = ovalbumin sensitized/challenged mice; Mon = ovalbumin sensitized/challenged mice treated with montelukast (30 mg/kg); **DK-1014** = ovalbumin sensitized/challenged mice treated with **DK-1014** (30 mg/kg). ^##^p < 0.005 compared to the normal control; **p < 0.005 compared to OVA mice.

The lung tissue of asthmatic mice exhibited extensive airway inflammation following H&E staining for peribronchiolar and perivascular lesions. However, **DK-1014**-treated mice exhibited less inflammatory cell infiltration into the peribronchiolar and perivascular lesions compared to OVA mice (Fig. 6a). OVA mice exhibited an overproduction of mucus under PAS staining whereas sections from **DK-1014**-treated mice exhibited a significant reduction in mucus hyperproduction in the airway compared to asthmatic mice (Fig. 6b).

## Conclusion

In summary, we designed and synthesized a series of homoegonol analogues (**8–22**). Among the synthesized compounds, homoegonol **8**, phenyl analogue **22a**, and 3,5-fluorophenyl analogue **DK-1014** exhibited excellent IL-6 suppression and moderate NO inhibition in Raw264.7 cells. Compound **DK-1014** exhibited more potent inhibitory activity of IL-6 (IC_50_ 16.19 ± 0.20 μM) than homoegonol **8** (IC_50_ = 19.94 ± 1.26 μM). Therefore, we investigated the anti-inflammatory effects of **DK-1014** in PMA-treated A549 cells in more detail.

Inflammation is a complex reaction against many pathological conditions including tissue injury and these inflammatory responses have critical functions in host immune defense, signal transduction pathways, and airway regulation. In this study, PMA-treated A549 cells exhibited an increase in IL-6, IL-8 and MCP-1 protein and IL-6 and IL-8 (containing MMP-9) mRNA expression whereas their expression in DK-1014-treated cells was lower in a dose-dependent manner (Fig. 1).

MAPKs have a major influence on the expression of inflammatory mediators^33^. Previous research has shown that IL-8 activates ERK and p38 but not JNK^34^, and our data indicates that PMA activates ERK and p38. In contrast, pretreatment with **DK-1014** inhibited the phosphorylation of p38 and ERK but not JNK (data not shown) and significantly downregulated the translocation of c-fos more than c-jun. This inhibition appears to be associated with **DK-1014**’s effect to inactivate multiple cellular transcription factors and signaling proteins such as AP-1, p38, and ERK (Fig. 2).

Previous studies have demonstrated that airway inflammation is suppressed by reducing the activation of the mTOR pathway in asthma^35^ and that the AKT/mTOR/p70S6K/GSK pathways play an important role in regulating the cell cycle of lung epithelial cells^36^. Therefore, we explored the effects of DK-1014 on the AKT pathway and found that DK-1014 suppressed the phosphorylation of downstream effectors (i.e., mTOR, p70S6K and GSK) and AKT phosphorylation. Additionally, DK-1014 strongly alleviated AKT phosphorylation more than LY294002^34^ and AS605240^37^ (a selective PI3Kgamma inhibitor) did, based on the results of immunofluorescent imaging (Fig. 3c). Together, this data suggests that the effect of DK-1014 contributes to the regulation of several complex regulatory pathways in PMA-treated A549 cells.

Asthma is chronic disease accompanied by airway inflammation and airway hyperresponsiveness as a result of various mechanisms. Of the various inflammatory cells, eosinophils in particular have cytotoxic proteins, lipid mediators, and cytokines which damage epithelial cells, lead to mucus hyperplasia, and cause AHR. In our animal study, the administration of DK-1014 effectively suppressed airway hyper-responsiveness (Fig. 4b), the production of Th2 cytokines and inflammatory cells in BALF, IgE production in serum (Fig. 5), the infiltration of inflammatory cells, and mucus overproduction (Fig. 6) compared with OVA-sensitized/challenged mice.

**Figure 7:**
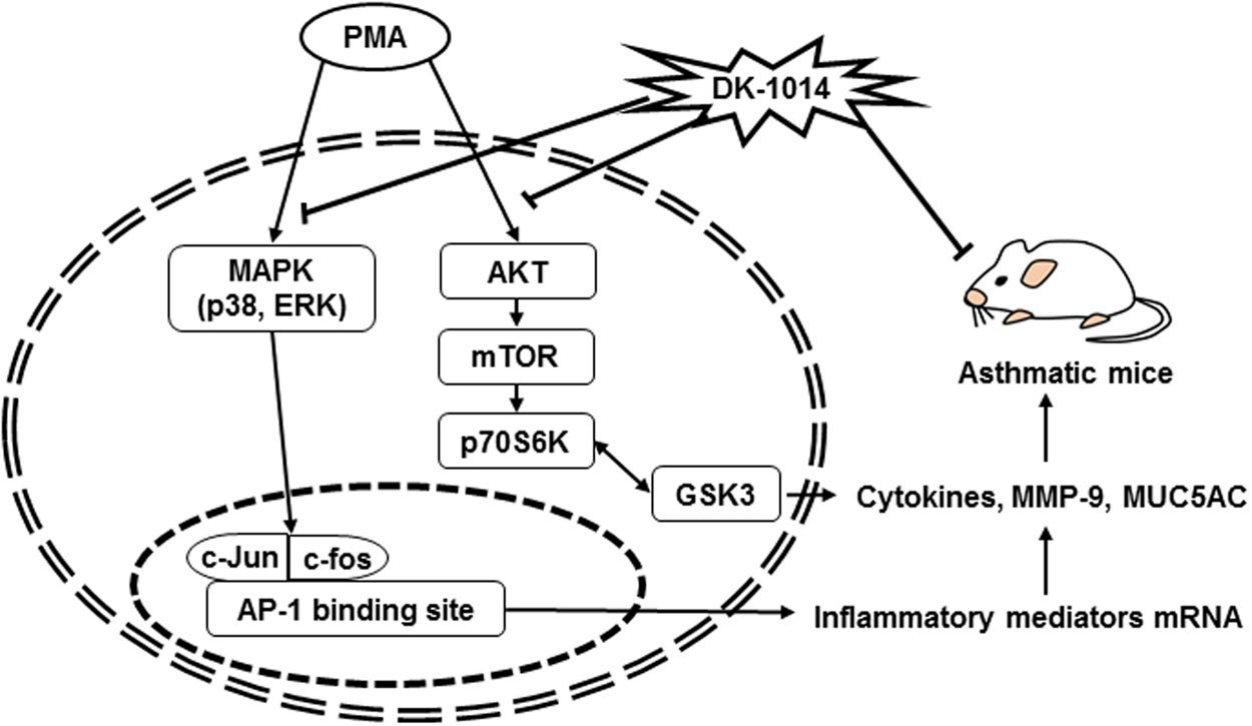
Proposed mechanism of **DK-1014** in PMA-treated lung epithelial cells and asthmatic mice. PMA activates the phosphorylation of MAPK (p38 and ERK) and AKT/mTOR/p70S6K/GSK3, which in turn AP-1 activation and eventually induces the mRNA and protein expression of inflammatory mediators associated with inflammatory cytokines, MMP-9, and MUC5AC. Downregulation of these inflammatory mediators by **DK-1014** leads to attenuation the recruitment of inflammatory cells, AHR, and the production of inflammatory cytokine and mucus in asthmatic mice (↓; activation, **⊥**; inhibition).

These anti-asthmatic effects of DK-1014 might be illustrated by inhibitory mechanism on mRNA and protein expression of inflammatory mediators (cytokines, MMP-9 and MUC5AC), which blocking the translocation of AP-1 and the phosphorylation of MAPK and AKT/mTOR/p70S6K/GSK3 in PMA-treated lung epithelial cells (Fig. 7).

This study thus indicates that **DK-1014** has a significant effect on pro-inflammatory cytokines, provides a molecular/biochemical basis for its anti-inflammatory actions, and suggests future avenues for drug discovery and development in the treatment of various inflammatory diseases, including asthma.

## Material and Methods

### Synthesis

All commercial chemicals were of reagent grade and were used without further purification. Solvents were dried using standard procedures. All reactions were performed under dried argon atmosphere in flame-dried glassware. Proton nuclear magnetic resonance (^1^H-NMR) spectra were analyzed using a Varian (400 MHz) spectrometer (Varian Medical Systems, Inc., Palo Alto, CA, USA), while ^13^C-NMR spectra were recorded using a Varian (100 MHz) spectrometer. Chemical shifts were provided in parts per million (ppm) downfield with coupling constants in hertz (Hz). Mass spectra were recorded using high resolution mass spectrometry (HRMS; electron ionization MS) obtained on a JMS-700 mass spectrometer (Jeol, Japan) or HRMS (electrospray ionization MS) obtained on a G2 QTOF mass spectrometer. The products from all reactions were purified by flash-column chromatography using silica gel 60 (230–400 mesh Kieselgel 60). Thin-layer chromatography on 0.25-mm silica plates (E. Merck; silica gel 60 F254) was used to monitor the reactions. Final product purity was determined using reversed-phase high pressure liquid chromatography (RP*-*HPLC), performed on a Waters Corp. HPLC system equipped with an ultraviolet (UV) detector at 254 nm. The mobile phases used were (A) H_2_O containing 0.05% trifluoroacetic acid and (B) CH_3_CN. The HPLC employed a YMC hydrosphere C18 (HS-302) column (5-µm particle size, 12-nm pore size) with a diameter of 4.6 mm and length of 150 mm at a flow rate of 1.0 ml/min. Compound purity was assessed using method A with a gradient of 25% B to 100% B in 35 min. All of the biologically evaluated compound purities were >95% using method A. Details of the synthetic schemes, methods, and characterization of the compounds are described in Supplementary Information.

### Cell culture

Human lung epithelial A549 cells and murine macrophage Raw 264.7 cells were purchased from ATCC (Rockville, MD). The cells were maintained at 37 °C in a humidified incubator containing 5% CO_2_ in Dulbecco’s modified Eagle’s medium (Welgene, Korea) or RPMI containing 10% fetal bovine serum (GibcoBRL, Invitrogen) and supplemented with 100 unit/ml penicillin and 100 μg/ml of streptomycin (GibcoBRL, Invitrogen). Cells were seeded onto culture plates and adhered for several hours. Cells were stimulated with LPS or PMA after preincubation with the various compounds (20 mM in DMSO) which was diluted with 1X PBS. Vehicle control cells was treated with 0.1% DMSO in PBS. Cells and the supernatant were harvested and stored at −80 °C until used.

### Cell viability assay

We used colorimetric MTT assays to study the effects of benzofuran derivatives on cell proliferation. Cells (1 × 10^4^ cells/well) were seeded onto a 96-well plate and treated with various concentrations of the compounds for 24 h. MTT (3-(4,5-dimethylthiazol-2-yl)-2,5-diphenyl tetrazolium bromide, Amresco, OH, USA) was dissolved in PBS at 5 mg/ml then added to the well plate and incubated for 4 h at 37 °C. The supernatant was suctioned, and formazan crystals were dissolved in 100 μl DMSO. Optical density was measured using a microplate reader (SPARK10, TECAN, Switzerland) at 570 nm and cell viability was expressed as the proportion of untreated cells.

### Nitric oxide assays

We used the modified Griess reaction to estimate the nitric oxide content in the cell culture supernatant^38^. The cells (5 × 10^4^ cells/well) were treated with various concentrations of the compound for 1 h followed by incubation with 0.5 μg/ml LPS for 24 h. The supernatant was mixed with the same volume of Griess reagent (1% sulfanilamide and 0.1% *N*-[1-naphthyl]-ethylenediamine dihydrochloride in 5% phosphoric acid, Sigma) and reacted at room temperature for 10 min. Optical density was measured at 540 nm with a microplate reader and LPS-induced Raw264.7 cells using a sodium nitrite standard.

### RT-PCR analysis

Total RNA was extracted from A549 cells using Trizol reagent (Invitrogen, CA, USA). The concentration and purity of the RNA were measured with a NanoDrop^TM^ 2000c spectrophotometer (ThermoFisher Scientific, Inc., Waltham, MA). Equal amounts of mRNA were converted by reverse-transcription into cDNA using a QuantiTect Reverse Transcription kit (Qiagen, Hilden, Germany). Polymerase chain reaction (PCR) analysis was conducted using specific primers and a premix (Fermentas, Germany) according to the manufacturer’s protocols, with the following PCR conditions: pre-denaturation at 94 °C for 5 min, 94 °C for 30 s, 60 °C for 30 s, and 72 °C for 45 s for 30 cycles and then a final extension phase at 72 °C for 10 min. GAPDH expression was included as an internal standard. PCR products were separated by electrophoresis on 1.5% agarose gel and visualized with a UV transilluminator (CoreBioSystem, Seoul, Korea). Images were captured with an Olympus C4000 camera (Olympus America Inc., Melville, NY, USA) and RT-PCR band intensities were measured using ImageJ software.

### Luciferase reporter assays

We used a dual luciferase reporter assay system (Promega, WI, USA) to analyze promoter activity. Cells were seeded into 48-well plates and incubated at 37 °C at 70–80% confluence. The cells were then incubated with serum-free media for 6 h and transiently co-transfected with an AP-1 promoter luciferase construct and pRL-SV plasmid (Promega, WI, USA) using LipofectAMINE 2000 reagent (Invitrogen, CA, USA) according to the manufacturer’s instructions. The medium was replaced with a basal medium after 5 h. The cells were lysed and luciferase activity was measured using SPARK10M. The luciferase signal for each sample was normalized to Renilla luciferase activity and expressed relative to the control.

### Western blot analysis

Western blot analysis was conducted as described previously^9^. Proteins were isolated using NP-40 (ELPIS-Biotech. Inc., Daejeon, Korea) at a concentration determined with a Pierce BSA kit. Equal amount of protein was resolved using sodium dodecyl sulfate polyacrylamide gel electrophoresis (SDS-PAGE). Proteins were transferred to a polyvinylidene difluoride (PVDF, Millipore) membrane, incubated for 1 h in 5% skim milk in a TBS-T buffer, then incubated with primary antibodies, including phospho and total ERK, AKT, GSK, 70S6K (1:1000 dilution; Cell signaling, Danvers MA), phospho and total p38 (1:500 dilution; Santacruz, Dallas, TX) and alpha-tubulin (1:1000 dilution; Millipore, Boston, MA). The membrane was washed with TBS-T buffer and incubated with secondary antibodies; goat anti-mouse (Santacruz), goat anti-rabbit (Jackson ImmunoResearch Laboratories) for 1 h. Each protein was detected using an enhanced chemiluminescence (ECL) detection system (Bio-Rad Laboratories, Inc.) following the manufacturer’s protocol. Western blot band intensities were measured using ImageJ software.

### Immunocytochemical analysis

A549 cells were adhered to chamber slides and treated with chemicals. The cells were immobilized in cold ethanol for 10 min and then washed with cold PBS three times. Slides were blocked with 2% (w/v) BSA in PBS and then incubated with anti-phospho-AKT overnight at 4 °C. The cells were subsequently washed with PBS and incubated with Alexa Fluor 488 coated anti-rabbit IgG secondary antibodies for 2 h at room temperature. After staining with Gold Antifade reagent containing DAPI (Invitrogen) for 5 min, the cells were washed and fluorescent images obtaining using LSM 510 m confocal microscopy (Carl Zeiss AG, Germany).

### Animal treatment

Specific pathogen-free female 6-week-old BALB/c mice were purchased from Koatech Co. (Pyeontaek, Korea) and used after 2 weeks quarantine and acclimatization in an air-conditioned room at approximately 22 °C and 55% RH. The mice were allowed sterilized tap water and standard rodent chow ad libitum. All experimental procedures were approved by the Institutional Animal Care and Use Committee of the Korea Research Institute of Bioscience and Biotechnology and were performed in compliance with National Institutes of Health Guidelines for the care and use of laboratory animals and Korean national animal welfare laws.

### Immunization and challenge

Mice were sensitized on days 1 and 14 with a 20-μg intraperitoneal (i.-p.-) injection of ovalbumin (OVA) emulsified in 2 mg of aluminium hydroxide in 200 μl of PBS buffer (pH 7.4). After initial sensitization, the mice received airway challenge with OVA (1%, w/v, in PBS) using an ultrasonic nebulizer (NE-U12; Omron Corp., Tokyo, Japan) for 1 h per day for days 21–23. DK-1014 and montelukast (Mon) were dissolved in dimethylacetamide and aliquoted into one dose. Samples were prepared in PBS contatining 10% Tween-80 and sonicated for 3 min fresh daily before each treatment and were administrated daily for days 21–23 by oral gavage (30 mg/kg) using feeding needle. Normal control mice (NC) were sensitized and challenged with PBS without ovalbumin and positive control mice were administered Mon by oral gavage (30 mg/kg)^23^.

### Airway hyperresponsiveness measurement

Twenty-four hours after the final OVA challenge, AHR was measured in conscious and unrestrained mice through whole-body plethysmography (OCP3000; Allmedicus, Seoul, Korea). Each mouse was placed in a plastic chamber and exposed to methylcholine aerosols at increasing concentrations (12.5–50 mg/mL in PBS) for 3 min. After each methylcholine challenge, enhanced pause (Penh) values were measured over 3 min. The results were expressed as the percentage Penh increase following each methylcholine dose, where the baseline Penh after PBS challenge was expressed as 100%^23^.

### BAL fluid collection and leukocyte count

Forty-eight hours after the final OVA challenge, mice were anesthetized via an intraperitoneal pentobarbital injection (50 mg/kg; Hanlim Pharm. Co., Seoul, Korea) and bronchoalveolar lavage fluid (BALF) was collected via lavage of the lung through the trachea with 0.4 ml of ice-cold PBS three times. The total number of inflammatory cells was measured with a hematocytometer. BALF was centrifuged at 200 g for 5 min at 4 °C to determine differential cell counts. The cell pellet was resuspended in PBS and centrifuged onto a slide using Cytospin (Hanil Science Industrial, Seoul, Korea). The slides were dried and cells stained with a Diff-Quik staining kit (B4132-1A; IMEB Inc., Deerfield, IL, USA). Supernatant from the BALF was stored at −70 °C for subsequent cytokine and IgE analysis.

### Cytokine and IgE assays

Enzyme linked immunosorbent assay (ELISA) kits were used to measure the inhibitory effects of the compounds on cytokine production. Cytokines were analyzed following the manufacturer’s instructions.

### Histology

After BALF sampling, lung tissue was excised from the mice and fixed with 10% (v/v) neutral formaldehyde solution for 24 h at room temperature. Fixed tissues were embedded in paraffin and then cut into 4-μm sections using a microtome (Leica, Nussloch, Germany). The resulting slides were stained with hematoxylin (MHS-16, Sigma) and eosin (HT110-1-32, Sigma) to estimate inflammatory cell infiltration into the peribronchial connective tissue. Periodic acid-Schiff (PAS, IMEB Inc., San Marcos, CA, USA) was used to evaluate mucus production in the lung tissue. Airway inflammation and mucus production were quantified using an image analyzer (Molecular Devices Ins., CA, USA).

### Statistical Analyses

All experiments were repeated at least three times and results were reported as means and standard deviations. Statistical significance was determined using a one-way analysis of variance (ANOVA) followed by a Tukey-Kramer test, with p < 0.05 as the standard for significance.

## Supplementary information

Novel benzofuran derivative DK-1014 attenuates the lung inflammation via blocking of MAPK/AP-1 and AKT/mTOR signaling in vitro and in vivo
