# Supplementary material for: Novel benzofuran derivative DK-1014 attenuates lung inflammation via blocking of MAPK/AP-1 and AKT/mTOR signaling in vitro and in vivo
Source: Sci Rep. 2019 Jan 29;9:862. doi: 10.1038/s41598-018-36925-9 (PMC6351650; doi:10.1038/s41598-018-36925-9)
Supplement: Supplementary file 1 — Novel benzofuran derivative DK-1014 attenuates the lung inflammation via blocking of MAPK/AP-1 and AKT/mTOR signaling in vitro and in vivo [file 41598_2018_36925_MOESM1_ESM.docx]

**Novel benzofuran derivative DK-1014 attenuates the lung inflammation via blocking of MAPK/AP-1 and AKT/mTOR signaling in vitro and in vivo**

**Xuezhen Xu^1†^, Ok-Kyoung Kwon^2,3†^, In-Sik Shin^4^, Jyotirling R. Mali^1^, Dipesh S. Harmalkar^1^, Yourim Lim^3^, Gilhye Lee^3^, Qili Lu^1^, Sei-Ryang Oh^3^, Kyung-Seop Ahn^3^, Hye-Gwang Jeong^2*^ & Kyeong Lee^1*^**

^1^ College of Pharmacy, Dongguk University-Seoul, Goyang-10326, Korea

^2^ Department of Toxicology, College of Pharmacy, Chungnam National University, Daejeon, 34134, Korea

^3^Natural Medicine Research Center, Korea Research Institute of Bioscience and Biotechnology, Chungju-si, Chungbuk 28116, Korea

**^4^** College of Veterinary Medicine, Chonnam National University, Gwangju, 61186, Korea

**Table of Contents**

Synthesis …………………………………………………………………..….………..3

Structure activity relationship study……..…..…………………………………..……..4

Experimental Section …………………………………………………………………..5

Scheme S1………………………………………………………………………………28

Scheme S2………………………………………………………………………………29

Scheme S3………………………………………………………………………………30

Scheme S4………………………………………………………………………………31

Scheme S5………………………………………………………………………………32

Scheme S6………………………………………………………………………………33

Scheme S7………………………………………………………………………………34

Fig S1……………………………………………………………………….…………..35

Fig S2……………………………………………………………………….…………..36

Fig S3……………………………………………………………………….…………..37

Fig S4………………………………………………………………………….………..38

**Synthesis.**

The key intermediates and final compounds were prepared following the synthetic pathways described in Schemes S1-7 (Supplementary Information). One of the major intermediates, 3,4-dimethoxyphenylacetylene **3**, was obtained by Corey-Fuchs reaction from 3,4-dimethoxybenzaldehyde **1**. First, 3,4-dimethoxybenzaldehyde was converted into dibromide **2** in 80% yield using Ramirez olefination. Treating dibromide **2** with lithium diisopropylamide (LDA) in tetrahydrofuran (THF) generated a bromoalkyne intermediate via dehydrohalogenation, which underwent subsequent lithium-halogen exchange followed by α-elimination to give alkyne **3** in 90% yield. Sonogashira coupling/cyclization of 5-iodovaniline **4** with 3,4-dimethoxyphenylacetylene **3** was conducted to give 2-(3,4-dimethoxyphenyl)-7-methoxybenzofuran-5-carbaldehyde **5** in good yield (70%). In our study we used 10% Pd/C, which is readily available and cheap, as a catalyst, along with PPh_3_ and CuI in aqueous conditions at 90°C. The HWE olefination of **5** with methyl (triphenylphosphoranylidene) acetate gave methoxybenzofuran acrylate **6** in 90% yield, followed by catalytic hydrogenation with Pd/C in acetic acid and THF at ambient temperature to give compound **7** in 95% yield. Compound **7** was reduced with LiAlH_4_ to afford homoegonol **8** in 95% yield (overall yield = 40.9 %**,** Scheme S1).

As shown in Scheme S2, acrylic ester **6** was subjected to catalytic hydrogenation with 10% Pd/C in THF at ambient temperature to give the ring-opened product **9** in 70% yield. The phenolic analogue **9** was then reduced using LiAlH_4_ to give 2-(3,4-dimethoxyphenethyl)-4-(3-hydroxypropyl)-6-methoxyphenol **10** in 90% yield (Scheme S2). The acyclic structure of compound **10** was confirmed using spectral analysis.

The synthesis of the methanol and ethanol analogues of homoegonol is described in Scheme S3. Aldehyde **5** was reduced with NaBH_4_ to give the desired methanol analogue **12**. For ethanol analogue **13**, compound **5** was subjected to Wittig olefination with methyltriphenylphosphonium iodide using sodium hydride in DMF at ambient temperature to afford vinyl benzofuran **11**, followed by hydroboration-oxidation with BH_3_ and basic hydrogen peroxide in THF gave compound **13** in 85% yield. The treatment of the hydroxyl group of **8** with acetyl chloride and triethylamine at ambient temperature gave *O*-acetyl compound **14** in 87% yield (Scheme S4). The acrylic ester analogue **6** underwent chemo-selective reduction with DIBAL-H gave the allylic alcohol compound **15** in 48% yield (Scheme S5). Compounds **16a** and **16b** were prepared by Wittig olefination of **5** with (carbethoxymethylene) triphenylphosphorane and triethyl 2-fluoro-2-phosphonoacetate, respectively, followed by reduction with DIBAL-H to yield the methyl prophenol compounds **17a** and **17b**, respectively (Scheme S6).

For homoegonol derivatives with various 2-aryl substituents **22a-q**, the Sonogashira coupling/cyclization of 5-iodovaniline **4** with alkynes **18a-q** in the presence of 10% Pd/C, PPh_3,_ and CuI in aqueous conditions was performed to give compounds **19a-q,** respectively, with 50-80% yields (Scheme S7). The synthesis of compounds **20a-q**, **21a-q**, and **22a-q** were achieved using a similar sequence for homoegonol **8**.

**Structure activity relationship study.**

Homoegonol **8** initially exhibited a very low inhibition of NO production (13.26±0.84%) but exhibited a potent IC_50_ value for IL-6 (19.94±1.26 μM) in Raw264.7 cells. Acyclic compound **10** exhibited moderate NO inhibition related to homoegonol **8** but did not decrease IL-6 production, indicating that the benzofuran backbone led to a higher inhibitory effect on IL-6.

When a hydroxyl alkyl chain was substituted at the R_1_ position, homoegonol **8** exhibited weaker NO inhibition and stronger IL-6 inhibitory activity related to the shorter side chain and *O*-acylated compounds such as **12**, **13**, and **14**. On the other hand, unsaturated side chain compounds **15**, **17a**, and **17b** reduced both the release of IL-6 and the inhibitory activity of NO production, suggesting that the R_1_ group, i.e. the hydroxylpropyl side chain, led to potent NO and IL-6 inhibition.

It was found that side-chain length and structural features based on the benzofuran skeleton had a significant effect on inhibitory potency. Accordingly, further modification focused on 2-aryl substitution in homoegonol moeity by replacing the R_2_ aryl substituent i.e., the dimethoxy group, with methoxy, fluorine, and trifluoromethyl groups. The compounds (**22a-e**) were evaluated for NO production inhibition and IL-6 activity in LPS-induced Raw264.7 cells. Amongst them, phenyl analogue **22a** and methoxyphenyl analogue **22d** exhibited good IC_50_ values for IL-6 (15.89±0.06, and 20.31±0.01 μM, respectively). Of note, compound **22a** was more potent than homoegonol **8** and 3-methoxyphenyl analogue **22d**. Therefore, we examined dimethoxy group substitution effects in the aryl ring of **8** on NO inhibition and IL-6 activity by replacing it with a fluoro group. NO inhibitory effects of the 3,5-difluorophenyl analogue, 22f, (named as DK-1014) and 3,4-difluorophenyl analogue, 22g, (30.36±9.82% and 93.32±1.88, respectively) and IC50 value for IL-6 (16.19±0.20 μM and 8.34±3.42, respectively) were higher than that for homoegonol 8 (13.26±0.84% and IC50 value for IL-6 = 19.94±1.26 μM), whereas compound 22g displayed higher cytotoxicity (8.34±3.42%). These findings suggest that the two fluoro groups at the *meta* position in the aryl ring enhance the inhibitory effects on NO and IL-6 production.

Following these results, we explored the effects on NO and IL-6 production of fluoro and trifluoromethyl substitution at the *o*, *m*, and *p*-positions in the phenyl ring (**22i-n**). Trifluoromethylphenyl analogue **22l** exhibited a higher NO inhibition (86.23±0.45%) and IC_50_ value for IL-6 (24.19±6.48 μM), indicating that having a CF_3_ group at the *ortho* position in the phenyl ring was more favorable for NO and IL-6 inhibition. However, compound **22l** exhibited the strongest cell viability (0.49±0.02%), and compounds **22o** and **22p** had lower NO and IL-6 inhibition. Compound **22q** also exhibited potent NO inhibition (90.12±2.03%) but did not improve the inhibitory effect of IL-6 at the same concentration. This indicates that dimethylamine, hydroxymethyl group installation, and ethereal linkage at the aryl ring had almost no observable effect on IL-6 inhibition.

**Experimental Section.**

**General.**

All the commercial chemicals were reagent grade and were used without further purification. Solvents were dried with standard procedures. All the reactions were carried out under an atmosphere of dried argon in flame-dried glassware. The proton nuclear magnetic resonance (^1^H-NMR) spectra were determined on a Varian (400 or 500 MHz) spectrometer (Varian Medical Systems, Inc. Palo Alto, CA, USA). ^13^C-NMR spectra were recorded on a Varian (100 or 125 MHz) spectrometer. The chemical shifts are provided in parts per million (ppm) downfield with coupling constants in hertz (Hz). The mass spectra were recorded using high-resolution mass spectrometry (HRMS) (electron ionization MS) obtained on a JMS-700 mass spectrometer (Jeol, Japan) or using HRMS (electrospray ionization MS) obtained on a G2 QTOF mass spectrometer. The products from all the reactions were purified by flash column chromatography using silica gel 60 (230-400 mesh Kieselgel 60). Additionally, thin-layer chromatography on 0.25-mm silica plates (E. Merck; silica gel 60 F254) was used to monitor reactions. The purity of the final products was checked by reversed phase high-pressure liquid chromatography (RP*-*HPLC), which was performed on a Waters Corp. HPLC system equipped with an ultraviolet (UV) detector set at 254 nm. The mobile phases used were (A) H_2_O containing 0.05% trifluoroacetic acid, and (B) CH_3_CN. HPLC employed an YMC Hydrosphere C18 (HS-302) column (5-µm particle size, 12-nm pore size) that was 4.6 mm in diameter × 150 mm in size with a flow rate of 1.0 mL/min. The compound purity was assessed either using (Method A) a gradient of 20% B to 100% B in 35 min or (Method B) a gradient of 25% B to 100% B in 35 min.

**4-(2,2-Dibromovinyl)-1,2-dimethoxybenzene (2).**

To a mixture of PPh_3_ (20.95 g, 80.0 mmol), CBr_4_ (6.62 g, 20.0 mmol) in dichloromethane (20 mL) was added 3,4-dimethoxybenzaldehyde **1** (1.60 g, 10.0 mmol) dropwise at 0 ˚C, then the reaction mixture was stirred at room temperature for 30 minutes and quenched with water. The mixture was extracted with dichloromethane and washed with brine. The organic layers were dried over anhydrous MgSO_4_, filtered and concentrated under *vacuo*. The resulting residue was purified by silica gel column chromatography (Hexanes:EtOAc = 9:1) to give 4-(2,2-dibromovinyl)-1,2-dimethoxybenzene as a yellow oil (3.72 g, 95.9% yield). ^1^H-NMR (CDCl_3,_ 400 MHz) *δ* 7.41 (1 s, H), 7.18 (d, *J* = 2.0 Hz, 1H), 7.09 (1 dd, *J* = 1.4 Hz, 8.8 Hz, 1H), 6.85 (d, *J* = 8.0 Hz, 1H), 3.89 (s, 3H), 3.88 (s, 3H).

**4-Ethynyl-1,2-dimethoxybenzene (3).**

To a solution of 4-(2,2-Dibromovinyl)-1,2-dimethoxybenzene **2** (3.72 g, 10.0 mmol) in dry tetrahydrofuron (16 mL) was added LDA (29 mL, 60.0 mmol) dropwise at -80 ˚C, then the mixture was stirred at same teperature for 2 h. After completion of the reaction, the reaction mixture was quenched with NH_4_Cl solution and extracted with dichloromethane. The organic layers were washed with brine and dried over anhydrous MgSO_4_. The mixture was filtered and concentrated in vacuum and the crude product was purified by column chromatography on silica gel column chromatography (Hexanes:EtOAc = 8:2) to give 4-ethynyl-1,2-dimethoxybenzene as yellow solid (1.70 g, 90.9% yield). ^1^H-NMR (CDCl_3,_ 400 MHz) *δ* 7.26 (s, 1H), 7.11 (1 d, *J* = 1.6 Hz, 4.8 Hz, H), 6.99 (d, *J* = 2.0 Hz, 1H), 6.81 (1 d, *J* = 5.6 Hz, 1H), 3.89 (3 s, H), 3.87 (s, 3H).

**2-(3,4-Dimethoxyphenyl)-7-methoxybenzofuran-5-carbaldehyde (5).**

To a 50.0 mL sealed tube equipped with a stir bar was added 5-iodovaniline **4** (0.30g, 1.08 mmol), 10 % Pd/C (0.03g, 0.03 mmol), PPh_3_ (0.03g, 0.13 mmol), CuI (0.01g, 0.06 mmol), L-prolinol or Et_3_N (0.32g or 0.32g, 3.00 mmol) and water, degassed with argon for about 1 hour. The 4-Ethynyl-1,2-dimethoxybenzene **5** (0.43g, 2.70 mmol) was added degassed continued for another 15 minutes, then the mixture was refluxed for 24 h. After completion of the reaction, the reaction mixture was partitioned between EtOAc and brine. The organic layer was separated, washed with water, dried over anhydrous MgSO_4_, filtered, and concentrated under *vacuo*. The resulting residue was purified by silica gel column chromatography (Hexanes:EtOAc = 7:3) to give 2-(3,4-Dimethoxyphenyl)-7-methoxybenzofuran-5-carbaldehyde as a yellow solid (0.36g, 62.5% yield). ^1^H-NMR (CDCl_3,_ 400 MHz) *δ* 10.01 (s, 1H), 7.70 (1 d, *J* = 1.2 Hz, 1H), 7.49 (dd, *J* = 2.0 Hz, 8.4 Hz, 1H), 7.38 (d, *J* = 2.0 Hz, 1H), 7.36 (1 s, 1H), 6.99 (s, 1H), 6.95 (d, *J* = 8.8 Hz, 1H), 4.10 (s, 3H), 4.00 (s, 3H), 3.95 (s, 3H).

**(*E*)-Methyl 3-[2-(3,4-dimethoxyphenyl)-7-methoxybenzofuran-5-yl]acrylate (6).**

To a solution of 2-(3,4-Dimethoxyphenyl)-7-methoxybenzofuran-5-carbaldehyde **5** (0.10g, 0.32 mmol) in dichloromethane (5 mL) was added methyl(triphenylphosphoranylidene)acetate (1.07 g, 3.20 mmol), then the reaction mixture was refluxed for 12 h. After completion of the reaction, the reaction mixture was concentrated under reduced pressure. The resulting residue was purified by silica gel column chromatography (Hexanes:EtOAc = 8:2) to give (*E*)-Methyl 3-[2-(3,4-dimethoxyphenyl)-7-methoxybenzofuran-5-yl]acrylate as white solid (0.10g, 85.5% yield). ^1^H-NMR (CDCl_3,_ 400 MHz) *δ* 7.77 (d, *J* = 16.0 Hz,1H), 7.47 (dd, *J* = 2.0 Hz, 8.4 Hz, 1H), 7.37 (d, *J* = 2.0 Hz, 1H), 7.33 (s, 1H), 6.97 (s, 1H), 6.94 (d, *J* = 1.2 Hz, 1H), 6.90 (s, 1H), 6.42 (d, *J* = 15.6 Hz, 1H), 4.08 (s, 3H), 3.99 (s, 3H), 3.94 (s, 3H), 3.83 (s, 3H).

**Methyl-3-(2-(3,4-dimethoxyphenyl)-7-methoxybenzofuran-5-yl)propanoate (7).**

To a solution of (*E*)-Methyl 3-[2-(3,4-dimethoxyphenyl)-7-methoxybenzofuran-5-yl]acrylate **6** (0.05g, 0.14 mmol) in THF (5 mL) was added Pd/C (0.03g, 0.014 mmol, 10 wt %), followed by one or two drops of CH_3_COOH and bubbled with hydrogen at room temperature for about 30 minutes under stirring. After completion of the reaction, the reaction mixture was concentrated under reduced pressure. The resulting residue was purified by silica gel column chromatography (Hexanes:EtOAc = 7:3) to give methyl 3-(2-(3,4-dimethoxyphenyl)-7-methoxybenzofuran-5-yl)propanoate as white solid (0.05g, 95.4% yield). ^1^H-NMR (CDCl_3,_ 400 MHz) *δ* 7.45 (dd, *J* = 2.0 Hz, 8.8 Hz, 1H), 7.36 (d, *J* = 2.0 Hz, 1H), 6.99 (s, 1H), 6.92 (d, *J* = 8.0 Hz, 1H), 6.83 (s, 1H), 6.63 (s, 1H), 4.03 (s, 3H), 3.98 (s, 3H), 3.93 (s, 3H), 3.68 (s, 3H), 3.02 (t, *J* = 8.0 Hz, 1H), 2.68 (d, *J* = 7.8 Hz, 1H).

**3-[2-(3,4-Dimethoxyphenyl)-7-methoxybenzofuran-5-yl]propan-1-ol (Homoegonol, 8).**

To a solution of methyl 3-(2-(3,4-dimethoxyphenyl)-7-methoxybenzofuran-5-yl)propanoate **7** (0.06g, 0.15 mmol) in THF (5 mL) was added LiAlH_4_ in THF (2.0 M, 0.15 mL, 0.31 mmol) dropwise at 0 ˚C, then the mixture was stirred at room temperature until the reaction was complete as judged by TLC. The reaction mixture was then acidified with 10% HCl and then partitioned between EtOAc and brine. The organic layer was separated, dried over anhydrous MgSO_4_, filtered, and concentrated under *vacuo*. The resulting residue was purified by silica gel column chromatography (Hexanes:EtOAc = 5:5) to give 3-[2-(3,4-Dimethoxyphenyl)-7-methoxybenzofuran-5-yl]propan-1-ol as a white solid (0.05g, 87.5% yield). ^1^H-NMR (CDCl_3,_ 400 MHz) *δ* 7.45 (dd, *J* = 2.0 Hz, 8.5 Hz, 1H), 7.37 (d, *J* = 2.0 Hz, 1H), 6.98 (d, *J* = 1.2 Hz, 1H), 6.93 (d, *J* = 8.4 Hz, 1H), 6.84 (s, 1H), 6.64 (d, *J* = 1.2 Hz, 1H), 4.04 (s, 3H), 3.99 (s, 3H), 3.93 (s, 3H), 3.72 (q, *J* = 5.6 Hz, 2H), 2.79 (t, *J* = 8.0 Hz, 2H), 1.92-1.99 (m, 2H); ^13^C-NMR (CDCl_3,_ 125 MHz) *δ* 148.7, 147.1, 146.2, 141.6, 135.1, 132.7, 127.3, 122.2, 120.3, 112.0, 111.2, 108.7, 62.3, 35.6, 34.5, 32.3, 31.8, 29.7; HRMS [M + H]+ calcd [C_20_H_23_O_5_], 343.1545; found, 343.1537; Purity 99.99% (as determined by RP-HPLC, method A, *t*_R_ = 14.45 min).

**Methyl-3-(3-(3,4-dimethoxyphenethyl)-4-hydroxy-5-methoxyphenyl)propanoate (9).**

To a solution of (*E*)-Methyl 3-[2-(3,4-dimethoxyphenyl)-7-methoxybenzofuran-5-yl]acrylate **6** (0.01g) in THF (5 mL) was added Pd/C (0.5 equiv., 10 wt %), stirred for 10 h under hydrogen atmosphere at room temperature. After completion of the reaction, the reaction mixture was filtered through a Celite pad and concentrated under reduced pressure. The resulting residue was purified by silica gel column chromatography (Hexanes:EtOAc = 7:3) to give Methyl-3-(3-(3,4-dimethoxyphenethyl)-4-hydroxy-5-methoxyphenyl)propanoate as a white solid (0.098g, 98.4% yield) ). ^1^H-NMR (CDCl_3,_ 400 MHz) *δ* 6.74-6.80 (m, 2H), 6.71 (d, *J* = 2.0 Hz, 1H), 6.58 (d, *J* = 2.0 Hz, 1H), 6.53 (d, *J* = 1.6 Hz, 1H), 5.58 (s, 1H), 3.87 (s, 3H), 3.86 (s, 3H), 3.85 (s, 3H), 3.67 (s, 3H), 2.82-2.89 (m, 6H), 2.57 (d, *J* = 7.8 Hz, 1H).

**2-(3,4-Dimethoxyphenethyl)-4-(3-hydroxypropyl)-6-methoxyphenol (10).**

To a solution of Methyl-3-(3-(3,4-dimethoxyphenethyl)-4-hydroxy-5-methoxyphenyl)propanoate **9** (0.08g equiv.) in THF (10 ml) was added LiAlH_4_ in THF (2.0 M, 1.0 equiv) dropwise at 0 ˚C, then the mixture was stirred at room temperature until the reaction was complete as judged by TLC. The reaction mixture was then acidified with 10% HCl and then partitioned between EtOAc and brine. The organic layer was separated, dried over anhydrous MgSO_4_, filtered, and concentrated under *vacuo*. The resulting residue was purified by silica gel column chromatography (Hexanes:EtOAc = 7:3) to obtain 2-(3,4-dimethoxyphenethyl)-4-(3-hydroxypropyl)-6-methoxyphenol as a White solid (0.068g, 91.2% yield). ^1^H-NMR (CDCl_3,_ 500 MHz) *δ* 7.45 (dd, *J* = 2.0 Hz, 8.5 Hz, 1H), 7.37 (d, *J* = 2.0 Hz, 1H), 6.98 (s, 1H ), 6.93 (d, *J* = 8.5 Hz, 1H), 6.84 (s, 1H), 6.64 (d, *J* = 0.5 Hz, 1H), 4.04 (s, 3H), 3.99 (s, 3H), 3.93 (s, 3H), 3.72 (t, *J* = 6.5 Hz, 2H), 2.79 (t, *J* = 7.5 Hz, 2H), 1.93-1.98 (m, 2H); ^13^C-NMR (CDCl_3,_ 125 MHz) *δ* 156.4, 149.6, 149.2, 144.8, 137.5, 131.2, 123.6, 118.8, 112.3, 111.4, 108.3, 107.3, 100.3, 62.3, 56.1, 56.0, 34.7, 32.4; HRMS [M + H]^+^ calcd [C_20_H_26_O_5_], 347.1858; found, 347.1855; Purity 99.99% (as determined by RP-HPLC, method A, *t*_R_ = 11.81 min).

**2-(3,4-Dimethoxyphenyl)-7-methoxy-5-vinylbenzofuran (11).**

To a mixture of 2-(3,4-Dimethoxyphenyl)-7-methoxybenzofuran-5-carbaldehyde **5** (60 mg, 0.19 mmol), methyltriphenylphosphonium iodide (116 mg, 0.29 mmol) in DMF (5 mL) was added sodium hydride (0.023 g, 0.95 mmol) at 0 ˚C, then the mixture was stirred for 12 h at room temperature. The reaction mixture was then partitioned between EtOAc and brine. The organic layer was separated, dried over anhydrous MgSO_4_, filtered, and concentrated under *vacuo*. The resulting residue was purified by silica gel column chromatography (Hexanes:EtOAc = 7:3) to give 2-(3,4-Dimethoxyphenyl)-7-methoxy-5-vinylbenzofuran as a white solid (0.05 g, 83.3% yield). ^1^H-NMR (CD_3_OD_,_ 500 MHz) *δ* 7.47 (dd, *J* = 2.0 Hz, 8.0 Hz, 1H), 7.44 (d, *J* = 2.0 Hz, 1H), 7.18 (s, 1H), 7.04 (d, *J* = 8.5 Hz, 1H), 7.02 (s, 1H), 6.98 (s, 1H), 6.79 (dd, *J* = 10.5 Hz, 17.5 Hz, 1H), 5.74 (d, *J* = 17.5 Hz,1H), 5.18 (d, *J* = 11.0 Hz, 1H), 4.04 (s, 3H), 3.93 (s, 3H), 3.88 (s, 3H).

**[2-(3,4-dimethoxyphenyl)-7-methoxybenzofuran-5-yl]methanol (12).**

To a solution of 2-(3,4-Dimethoxyphenyl)-7-methoxybenzofuran-5-carbaldehyde **5** (0.1 g, 0.32 mmol) in THF (5 mL) was added NaBH_4_ (0.036 g, 0.96 mmol) at 0 ˚C, then the mixture was stirred at room temperature until the reaction was complete as judged by TLC. The reaction mixture was then acidified with 10% HCl and then partitioned between EtOAc and brine. The organic layer was separated, dried over anhydrous MgSO_4_, filtered, and concentrated under *vacuo*. The resulting residue was purified by silica gel column chromatography (Hexanes:EtOAc = 6:4) to give [2-(3,4-dimethoxyphenyl)-7-methoxybenzofuran-5-yl]methanol as a white solid (0.09g, 84.6% yield).^1^H-NMR (CDCl_3,_ 500 MHz) *δ* 7.50 (dd, *J* = 2.0 Hz, 8.0 Hz, 1H), 7.47 (d, *J* = 1.5 Hz, 1H), 7.15 (s, 1H), 7.12 (s, 1H), 7.07 (d, *J* = 8.5 Hz, 1H), 6.92 (s, 1H), 4.68 (d, *J* = 6.0 Hz, 2H), 4.14 (t, *J* = 6.0 Hz, 1H), 4.02 (s, 3H), 3.92 (s, 3H), 3.87 (s, 3H); ^13^C-NMR (CDCl_3,_ 125 MHz) *δ* 156.7, 149.7, 149.2, 145.2, 143.5, 136.8, 131.1, 123.4, 118.2, 111.5, 111.4, 108.3, 105.8, 100.4, 65.9, 56.1, 56.0; HRMS [M + H]^+^ calcd [C_18_H_18_O_5_], 315.1232; found, 315.1223; Purity >99.98 % (as determined by RP-HPLC, method A, *t*_R_ = 12.37 min).

**2-[2-(3,4-Dimethoxyphenyl)-7-methoxybenzofuran-5-yl]ethanol (13).**

To a solution of 2-(3,4-Dimethoxyphenyl)-7-methoxy-5-vinylbenzofuran **11** (30 mg, 0.10 mmol) in THF (2mL) was added BH_3_·THF in THF (1.0 M, 0.11 mL, 0.11 mmol) dropwise at 0 ˚C, then the mixture was stirred at room temperature for 3 h. After 3 h of stirring at room temperature, added 0.1 mL of 10 % NaOH solution and 0.1 ml of 30 % H_2_O_2_ and stirred for 30 minutes at room temperature and at 55 ˚C for 1 h. The reaction mixture was then partitioned between EtOAc and brine. The organic layer was separated, dried over anhydrous MgSO_4_, filtered, and concentrated under *vacuo*. The resulting residue was purified by silica gel column chromatography (Hexanes:EtOAc = 5:5) to give 2-[2-(3,4-Dimethoxyphenyl)-7-methoxybenzofuran-5-yl]ethanol as a white solid (0.014 g, 43.8% yield). ^1^H-NMR (CD_3_OD_,_ 500 MHz) *δ* 7.46 (dd, *J* = 2.0 Hz, 8.5 Hz, 1H), 7.44 (d, *J* = 2.0 Hz, 1H), 7.03 (d, *J* = 8.0 Hz, 1H), 7.01 (s, 1H), 6.98 (s, 1H), 6.74 (d, *J* = 0.5 Hz, 1H), 4.01 (s, 3H), 3.92 (s, 3H), 3.87 (s, 3H), 3.79 (t, *J* = 7.0 Hz, 2H), 2.88 (t, *J* = 7.0 Hz, 2H); ^13^C-NMR (CDCl_3,_ 125 MHz) *δ* 156.5, 149.7, 149.2, 145.0, 134.0, 131.4, 123.4, 118.2, 113.1, 111.4, 108.3, 107.6, 100.2, 63.9, 56.2, 56.1, 56.0, 39.5; HRMS [M + H]^+^ calcd [C_19_H_20_O_5_], 329.1389; found, 329.1376; Purity 99.98 % (as determined by RP-HPLC, method A, *t*_R_ = 13.26 min).

**3-(2-(3,4-Dimethoxyphenyl)-7-methoxybenzofuran-5-yl)propyl acetate (14).**

To a solution of 3-(2-(3,4-dimethoxyphenyl)-7-methoxybenzofuran-5-yl)propan-1-ol **8** (0.5g, 1.46 mmol) in MC (15 mL) Acetyl chroride (0.229 g, 2.90 mmol) and TEA (0.455 g, 4.5 mmol were added at 0 ˚C, then the mixture was stirred at room temperature 12 hours. The reaction mixture was stopped by water then partitioned between EtOAc and brine. The organic layer was separated, dried over anhydrous MgSO_4_, filtered, and concentrated under *vacuo*. The resulting residue was purified by silica gel column chromatography (Hexanes:EtOAc = 8:2) to give 3-(2-(3,4-dimethoxyphenyl)-7-methoxybenzofuran-5-yl)propyl acetate as a white solid ( 0.49g, 87.5% yield). ^1^H-NMR (CDCl_3,_ 400 MHz) *δ* 7.45 (dd, *J* = 2.0 Hz, *J* = 2.0 Hz, 1H), 7.36 (d, *J* = 2.0 Hz, 1H), 6.96 (d, *J* = 1.2 Hz, 1H), 6.93 (d, *J* = 8.4 Hz, 1H), 6.84 (s, 1H), 6.61 (d, *J* = 1.2 Hz, 1H), 4.13 (d, *J* = 6.8 Hz, 2H), 4.04 (s, 3H), 3.99 (s, 3H), 3.93 (s, 3H), 2.76 (d, *J* = 7.6 Hz, 2H), 2.07 (s, 3H), 1.97-2.04 (m, 2H); ^13^C-NMR (CDCl_3,_ 100 MHz) *δ* 171.2, 156.4, 149.5, 149.1, 144.8, 142.8, 142.5, 136.9, 131.1, 123.5, 118.1, 112.3, 111.2, 108.1, 107.1, 100.3, 63.9, 56.1, 56.1, 56.0, 32.5, 30.7, 21.0; HRMS [M + H]^+^ calcd [C_22_H_24_O_6_], 385.1651; found, 385.1651; Purity 99.99% (as determined by RP-HPLC, method A, *t*_R_ = 21.61 min).

**(*E*)-3-(2-(3,4-Dimethoxyphenyl)-7-methoxybenzofuran-5-yl)prop-2-en-1-ol (15).**

To a solution of (*E*)-Methyl 3-[2-(3,4-dimethoxyphenyl)-7-methoxybenzofuran-5-yl]acrylate **6** (0.04g, 0.11 mmol) in THF (10ml) was added DIBAL-H in Toluene (1.0 M, 2.0 equiv) dropwise at 0 ˚C then the reaction mixture was stirred for 2 h at room temperature. After completion of the reaction, the reaction mixture was then acidified with 10% HCl and then partitioned between EtOAc and brine. The organic layer was separated, dried over anhydrous MgSO_4_, filtered, and concentrated under *vacuo*. The resulting residue was purified by silica gel column chromatography (Hexanes:EtOAc = 6:4) to give (*E*)-Methyl 3-[2-(3,4-dimethoxyphenyl)-7-methoxybenzofuran-5-yl]acrylate as a white solid (0.02g, 48% yield). ^1^H-NMR (CDCl_3,_ 400 MHz) *δ* 7.46 (dd, *J* = 1.8 Hz, 8.6 Hz, 1H), 7.36 (d, *J* = 1.6 Hz, 1H), 7.15 (s, 1H), 6.93 (d, *J* = 8.0 Hz, 1H), 6.87 (d, *J* = 1.2 Hz, 1H), 6.86 (s, 1H), 6.68 (d, *J* = 7.6 Hz,1H), 6.35 (dt, *J* = 5.9 Hz, 15.7 Hz, 1H), 4.35 (t, *J* = 1.6 Hz, 2H), 4.06 (s, 3H), 3.98 (s, 3H), 3.93 (s, 3H), 1.41 (t, *J* = 6.0 Hz, 1H); ^13^C-NMR (CDCl_3,_ 100 MHz) *δ* 156.7, 149.7, 149.2, 145.1, 143.8, 132.8, 131.9, 131.3, 127.3, 123.3, 118.2, 111.7, 111.3, 108.2, 104.6, 100.4, 63.9, 56.1, 56.0; HRMS [M + H]^+^ calcd [C_20_H_21_O_5_], 341.1389; found, 341.1389; Purity 99.99% (as determined by RP-HPLC, method A, *t*_R_ = 14.67 min).

(**E)-Ethyl 3-(2-(3,4-dimethoxyphenyl)-7-methoxybenzofuran-5-yl)-2-methylacrylate (16a).**

To a solution of 2-(3,4-dimethoxyphenyl)-7-methoxybenzofuran-5-carbaldehyde **5** (0.10g, 0.32 mmol) in dichloromethane (10ml) was added (carbethoxymethylene)triphenylphosphorane (0.23g, 0.64 mmol), then the reaction mixture was refluxed for 12 h. After completion of the reaction, the reaction mixture was concentrated under reduced pressure. The resulting residue was purified by silica gel column chromatography (Hexanes:EtOAc = 8:2) to give (E)-ethyl 3-(2-(3,4-dimethoxyphenyl)-7-methoxybenzofuran-5-yl)-2-methylacrylate as a white solid (0.10g, 83.8% yield). ^1^H-NMR (CDCl_3,_ 400 MHz) *δ* 7.77 (d, *J* = 1.2 Hz, 1H), 7.47 (d, *J* = 8.4 Hz, 1H), 7.37 (s, 1H ), 7.23 (s, 1H), 6.94 (d, *J* = 8.0 Hz, 1H), 6.91 (s, 1H), 6.82 (s, 1H), 4.29 (q, *J* = 6.9 Hz, 2H), 4.06 (s, 3H), 3.99 (s, 3H) 3.94 (s, 3H) 2.18 (s, 3H).

**(Z)-Ethyl 3-(2-(3,4-dimethoxyphenyl)-7-methoxybenzofuran-5-yl)-2-fluoroacrylate (16b).**

To a solution of 2-(3,4-dimethoxyphenyl)-7-methoxybenzofuran-5-carbaldehyde **5** (0.10g, 0.32 mmol) in THF (10ml) was added Triethyl 2-fluoro-2-phosphonoacetate (0.12g, 0.48 mmol), DBU (0.08g, 0.55 mmol) and LiCl (0.02g, 0.48 mmol) then the reaction mixture was stirred for 12 h. After completion of the reaction, the reaction mixture was stopped by water then partitioned between EtOAc and brine. The organic layer was separated, dried over anhydrous MgSO_4_, filtered, and concentrated under *vacuo*. The resulting residue was purified by silica gel column chromatography (Hexanes:EtOAc = 8:2) to give (Z)-ethyl 3-(2-(3,4-dimethoxyphenyl)-7-methoxybenzofuran-5-yl)-2-fluoroacrylate as a white solid (105mg, 82.7% yield). ^1^H-NMR (CDCl_3,_ 400 MHz) *δ* 7.46 (d, *J* = 8.8 Hz, 1H), 7.37 (s, 1H), 7.31 (s, 1H), 7.10 (s, 1H), 7.02 (t, *J* = 8.8 Hz, 1H), 6.95 (d, *J* =5.6 Hz, 1H), 6.92 (d, *J* =5.2 Hz, 1H), 6.88 (s, 1H), 4.28 (q, *J* = 14.0 Hz, 2H), 4.05 (s, 3H), 3.99 (s, 3H), 3.93 (s, 3H), 2.27 (t, *J* =6.8 Hz, 3H).

**(E)-3-(2-(3,4-dimethoxyphenyl)-7-methoxybenzofuran-5-yl)-2-methylprop-2-en-1-ol (17a).**

To a solution of (E)-ethyl 3-(2-(3,4-dimethoxyphenyl)-7-methoxybenzofuran-5-yl)-2-methylacrylate **16a** (0.07g, 0.18 mmol) in THF (10ml) was added DUBAL in Toluene (1.0 M, 2.0 equiv) dropwise at 0 ˚C then the reaction mixture was stirred for 2 h at room temperature. After completion of the reaction. The reaction mixture was then acidified with 10% HCl and then partitioned between EtOAc and brine. The organic layer was separated, dried over anhydrous MgSO_4_, filtered, and concentrated under *vacuo*. The resulting residue was purified by silica gel column chromatography (Hexanes:EtOAc = 6:4) to give (E)-3-(2-(3,4-dimethoxyphenyl)-7-methoxybenzofuran-5-yl)-2-methylprop-2-en-1-ol as a white solid (0.03g, 47% yield). ^1^H-NMR (CDCl_3,_ 400 MHz) *δ* 7.46 (dd, *J* _1_= 1.8 Hz, *J* _2_= 8.6 Hz, 1H), 7.37 (d, *J* = 2.0 Hz, 1H), 7.07 (s, 1H), 6.93 (d, *J* = 8.4 Hz, 1H), 6.87 (s, 1H), 6.70 (s, 1H), 6.60 (s, 1H), 4.22 (s, 2H), 4.04 (s, 3H), 3.98 (s, 3H), 3.93 (s, 3H), 2.04 (s, 3H), 0.83-0.89 (m, 1H); ^13^C-NMR (CDCl_3,_ 100 MHz) *δ* 156.5, 149.6, 149.1, 144.5, 142.7, 136.9, 133.4, 130.9, 125.6, 123.3, 118.1, 113.2, 111.2, 108.1, 107.8, 100.4, 69.1, 56.1, 56.0, 15.4. HRMS [M + H]^+^ calcd [C_21_H_23_O_5_], 355.1545; found, 355.1544; Purity 99.97% (as determined by RP-HPLC, method A, *t*_R_ = 16.02 min).

**(Z)-3-(2-(3,4-Dimethoxyphenyl)-7-methoxybenzofuran-5-yl)-2-fluoroprop-2-en-1-ol (17b).**

To a solution of (Z)-ethyl 3-(2-(3,4-dimethoxyphenyl)-7-methoxybenzofuran-5-yl)-2-fluoroacrylate **16b** (0.04g, 0.10 mmol) in THF (10ml) was added DIBAL in Toluene (1.0 M, 2.0 equiv) dropwise at 0 ˚C then the reaction mixture was stirred for 2 h at room temperature. After completion of the reaction. The reaction mixture was then acidified with 10% HCl and then partitioned between EtOAc and brine. The organic layer was separated, dried over anhydrous MgSO_4_, filtered, and concentrated under *vacuo*. The resulting residue was purified by silica gel column chromatography (Hexanes:EtOAc = 6:4) to give (Z)-3-(2-(3,4-dimethoxyphenyl)-7-methoxybenzofuran-5-yl)-2-fluoroprop-2-en-1-ol as a white solid (0.02g, 50% yield). ^1^H-NMR (CDCl_3,_ 400 MHz) *δ* 7.46 (dd, *J* = 2.0 Hz, 8.4 Hz, 1H), 7.37 (d, *J* = 2.0 Hz, 1H), 7.30 (s, 1H), 7.02 (s, 1H), 6.93 (d, *J* = 7.6 Hz, 1H), 6.88 (s, 1H), 6.85 (d, *J* = 38.4 Hz, 1H), 4.22 (dd, *J* = 6.6 Hz, 15.0 Hz, 2H), 4.05 (s, 3H), 3.99 (s, 3H), 3.93 (s, 3H), 1.84 (t, *J* = 6.2 Hz, 1H); NMR (CDCl_3,_ 100 MHz) *δ* 158.5, 156.7, 155.9, 149.6, 149.1, 144.8, 143.3, 131.1, 128.5, 128.4 123.2, 118.2, 113.8, 113.7, 111.2, 108.1, 108.1, 107.2, 107.2, 100.4, 62.3, 62.0, 56.1, 56.0. HRMS [M + H]^+^ calcd [C_20_H_20_O_5_F], 359.1295; found, 359.1296; Purity 99.96% (as determined by RP-HPLC, method A, *t*_R_ = 15.73 min).

**General procedure of Sonogashira reaction for synthesis of 19a-q.**

To a 50.0 mL sealed tube equipped with a stir bar was added 5-iodovaniline (1 equiv), 10 % Pd/C (0.03 equiv), PPh_3_ (0.12 equiv), CuI (0.06 equiv), L-prolinol or Et_3_N (3.0 equiv) and water, degassed with argon for about 1 hour. The acetylenecompound (2.5 equiv) was added degassed continued for another 15 minutes, then the mixture was refluxed for 24 h. After completion of the reaction, the reaction mixture was partitioned between EtOAc and brine. The organic layer was separated, washed with water, dried over anhydrous MgSO_4_, filtered, and concentrated under *vacuo*. The resulting residue was purified by silica gel column chromatography to obtain **19a-q**.

**7-Methoxy-2-phenylbenzofuran-5-carbaldehyde (19a).**

Yellow solid 0.350g, 82.8% yield.^1^H-NMR (CDCl_3_, 400 MHz) *δ* 10.01 (s, 1H), 7.91 (d, *J* = 8.4 Hz, 2H), 7.73 (s, 1H), 7.47 (t, *J* = 7.6 Hz, 2H), 7.41 (d, *J* = 7.6 Hz, 1H), 7.38 (s, 1H), 7.12 (s, 1H), 4.10 (s,, 3H).

**2-(3,5-Dimethoxyphenyl)-7-methoxybenzofuran-5-carbaldehyde (19b).**

Yellow solid 0.30 g, 53.4% yield. ^1^H-NMR (CDCl_3,_ 400 MHz) *δ* 10.01 (s, 1H), 7.72 (d, *J* = 0.8 Hz, 1H), 7.38 (s, 1H), 7.09 (s, 1H), 7.04 (d, *J* = 2.8 Hz, 2H), 6.51 (t, *J* = 2.2 Hz, 2H), 4.10 (s, 3H), 3.88 (s, 6H).

**7-Methoxy-2-(2-methoxyphenyl)benzofuran-5-carbaldehyde (19c).**

Yellow solid 0.35g, 69.1% yield. ^1^H-NMR (CDCl_3_, 400 MHz) δ 10.01 (d, *J* = 0.8 Hz, 1H), 8.13 (dd, *J* = 1.6, 7.6 Hz, 1H), 7.73 (d, *J* = 1.6 Hz, 1H), 7.43 (s, 1H), 7.37 (dd, *J* = 1.7, 8.1 Hz, 2H), 7.07-7.11 (m, 1H), 7.03 (d, *J* = 8.4 Hz, 1H), 4.10 (s, 3H), 4.02 (s, 3H).

**7-Methoxy-2-(3-methoxyphenyl)benzofuran-5-carbaldehyde (19d).**

Yellow solid 0.34g, 71.8% yield. ^1^H-NMR (CDCl_3_, 400 MHz) *δ* 10.01 (s, 1H), 7.72 (d, *J* = 1.5 Hz, 1H), 7.49 (d, *J* = 7.5 Hz, 1H), 7.43 (t, *J* = 2.0 Hz, 1H), 7.38 (t, *J* = 8.0 Hz, 2H), 7.11 (s, 1H), 6.95 (dd, *J* = 2.0 Hz, 8.0 Hz, 1H), 4.10 (s, 3H), 3.90 (s, 3H).

**7-Methoxy-2-(4-methoxyphenyl)benzofuran-5-carbaldehyde (19e).**

Yellow solid 0.40g, 80% yield.^1^H-NMR (CDCl_3_, 400 MHz) *δ* 10.00 (s, 1H), 7.83 (d, *J* = 9.2 Hz, 2H), 7.69 (d, *J* = 1.2 Hz, 1H), 7.34 (d, *J* = 0.8 Hz, 1H), 6.98 (d, *J* = 8.4 Hz, 2H) 6.96 (s, 1H), 4.09 (s, 3H), 3.87 (s, 3H).

**2-(3,5-Difluorophenyl)-7-methoxybenzofuran-5-carbaldehyde (19f).**

Yellow solid 0.07g, 54.1% yield. ^1^H-NMR (CDCl_3_, 400 MHz) *δ* 10.02 (s, 1H), 7.74 (s, 1H), 7.41 (d, *J* =4.8, 3H), 7.15 (s, 1H), 6.83 (t, *J* =8.8, 1H), 4.10 (s, 3H).

**2-(3,4-Difluorophenyl)-7-methoxybenzofuran-5-carbaldehyde (19g).**

Yellow solid 0.15g, 75.6% yield. ^1^H-NMR (CDCl_3_, 400 MHz) *δ* 10.02 (s, 1H), 7.69-7.74 (m, 2H), 7.75 (s, 1H), 7.40 (s, 1H), 6.94-7.04 (m, 2H), 4.10 (s, 3H).

**2-(2,4-Difluorophenyl)-7-methoxybenzofuran-5-carbaldehyde (19h).**

Yellow solid 0.17g, 78.5% yield. ^1^H-NMR (CDCl_3_, 400 MHz) *δ* 10.02 (s, 1H), 8.09 (q, *J* =8.4, 1H), 7.61-7.65 (m, 1H), 7.39 (s, 1H), 7.26 (q, *J* =8.4, 1H), 7.07 (s, 1H), 4.10 (s, 3H).

**2-(2-Fluorophenyl)-7-methoxybenzofuran-5-carbaldehyde (19i).**

Yellow solid (2.00g, 90.0 % yield). ^1^H NMR (CDCl_3_, 400 MHz) *δ* 10.02 (s, 1H), 8.10 (t, *J* = 7.8 Hz, 1H), 7.75 (s, 1H), 7.40 (s, 1H), 7.36 (t, *J* = 7.2 Hz, 1H), 7.33 (d, *J* = 3.2 Hz, 1H), 7.28 (d, *J* = 7.6 Hz, 1H), 7.20 (dd, *J* = 8.2 Hz, 11.4 Hz, 1H), 4.10 (s, 3H).

**2-(3-Fluorophenyl)-7-methoxybenzofuran-5-carbaldehyde (19j).**

Yellow solid 0.37g, 77.8% yield. ^1^H-NMR (CDCl_3_, 400 MHz) *δ* 10.02 (s, 1H), 7.74 (s,1H), 7.68 (d, *J* = 8.0 Hz,1H), 7.58-7.62 (m, 1H), 7.44 (q, *J* = 8.0 Hz, 1H), 7.41 (d, *J* = 8.0 Hz, 1H), 7.14 (s, 1H), 7.09 (s, 1H),4.10 (s, 1H).

**2-(4-Fluorophenyl)-7-methoxybenzofuran-5-carbaldehyde (19k).**

Yellow solid 0.30 g, 61.7% yield. ^1^H-NMR (CDCl_3_, 400 MHz) *δ* 10.01 (s, 1H), 7.89 (dd, *J* = 5.2 Hz, 8.8 Hz, 2H), 7.72 (d, *J* = 1.2 Hz, 1H), 7.38 (d, *J* = 1.2, 1H), 7.16 (t, *J* = 8.8, 2H), 7.05 (s, 1H), 4.09 (s, 3H).

**7-Methoxy-2-(2-(trifluoromethyl)phenyl)benzofuran-5-carbaldehyde (19l).**

Brown solid (0.43 g, 74.6 % yield). ^1^H NMR (CDCl_3_, 400 MHz) *δ* 10.03 (s, 1H), 7.92 (d, *J* = 8.0 Hz, 1H), 7.83 (d, *J* = 8.0 Hz, 1H), 7.78 (s, 1H), 7.67 (t, *J* = 7.6 Hz, 1H), 7.56 (t, *J* = 7.8 Hz, 1H), 7.42 (s, 1H), 7.15 (s, 1H), 4.09 (s, 3H).

**7-Methoxy-2-(3-(trifluoromethyl)phenyl)benzofuran-5-carbaldehyde (19m).**

Yellow solid 0.30g, 52.1% yield. ^1^H-NMR (CDCl_3_, 400 MHz) *δ* 10.02 (s, 1H), 8.14 (s, 1H), 8.07(d, *J* = 7.2 Hz, 1H), 7.75 (d, *J* = 1.6 Hz, 1H), 7.62 (q, *J* = 8.0 Hz, 1H), 7.41 (d, *J* = 1.2 Hz, 1H), 7.21 (s, 1H), 4.11 (s, 3H).

**7-Methoxy-2-(4-(trifluoromethyl)phenyl)benzofuran-5-carbaldehyde (19n).**

Yellow solid 0.25g, 73.0% yield. ^1^H-NMR (CDCl_3_, 400 MHz) δ 10.02 (s, 1H), 8.01 (d, *J* = 8.4, 1H), 7.75 (d, *J* = 0.8 Hz, 1H), 7.72 (d, *J* = 8.4, 2H), 7.41 (d, *J* = 1.2 Hz, 1H), 7.23 (s, 1H), 4.11 (s, 3H).

**2-(4-(Dimethylamino)phenyl)-7-methoxybenzofuran-5-carbaldehyde (19o).**

Yellow solid 0.24g, 45.8% yield.^1^H-NMR (CDCl_3_, 400 MHz) *δ* 9.99 (d, *J* = 0.8 Hz, 1H), 7.70 (d, *J* = 7.2 Hz, 2H), 7.66 (t, *J* = 1.2 Hz, 1H), 7.32 (d, *J* = 0.8 Hz, 1H), 6.87 (s, 1H), 6.76 (d, *J* = 8.8 Hz, 2H), 4.09 (s, 3H), 3.03 (s, 6H).

**2-(4-(Hydroxymethyl)phenyl)-7-methoxybenzofuran-5-carbaldehyde (19p).**

Yellow solid 0.21g, 54.6% yield.^1^H-NMR (CDCl_3_, 400 MHz) *δ* 10.01 (s, 1H), 7.91 (d, *J* = 8.4 Hz, 2H), 7.73 (d, *J* = 0.8 Hz, 1H), 7.48 (d, *J* = 8.0 Hz, 2H), 7.38 (s, 1H), 7.12 (s, 1H), 4.77 (d, *J* = 6.0 Hz,, 2H), 4.10 (s,, 3H), 1.74 (t, *J* = 6.0 Hz, 1H).

**7-Methoxy-2-(4-phenoxyphenyl)benzofuran-5-carbaldehyde (19q).**

Yellow solid 0.18g, 75.8% yield.^1^H-NMR (CDCl_3_, 400 MHz) *δ* 10.01 (s, 1H), 7.84-7.87 (m, 2H), 7.71 (d, *J* = 1.6 Hz, 1H), 7.36-7.40 (m, 3H), 7.15-7.18 (m, 1H), 7.06-7.09 (m, 4H), 7.02 (s, 1H), 4.09 (s, 3H), 1.74 (t, *J* = 6.0 Hz, 1H).

**General procedure of Wittig reaction for synthesis of 20a-q.**

To a solution of aldehyde (1.0 equiv.) in dichloromethane (10 volume) was added methyl(triphenylphosphoranylidene)acetate (3.0 equiv.), then the reaction mixture was refluxed for 12 h. After completion of the reaction, the reaction mixture was concentrated under reduced pressure. The resulting residue was purified by silica gel column chromatography to yielded white solid **20a-q**.

**(*E*)-Methyl 3-(7-methoxy-2-phenylbenzofuran-5-yl)acrylate (20a).**

Yellow solid 0.15g, 81.9% yield. ^1^H-NMR (CDCl_3_, 400 MHz) *δ* 7.89 (d, *J* = 7.2 Hz, 2H), 7.77 (d, *J* = 16.4 Hz, 1H), 7.45 (t, *J* = 7.6 Hz, 2H), 7.38 (d, *J* = 7.2 Hz, 1H), 7.35 (s, 1H), 7.02 (s, 1H), 6.98 (d, *J* = 1.2 Hz, 1H), 6.42 (d, *J* = 16.0 Hz, 1H), 4.08 (s, 3H), 3.82 (s, 3H).

**(*E*)-Methyl-3-(2-(3,5-dimethoxyphenyl)-7-methoxybenzofuran-5-yl)acrylate (20b).**

Yield white solid 0.11g, 58.5% yield. ^1^H-NMR (CDCl_3,_ 400 MHz) *δ* 7.77 (d, *J* = 16 Hz, 1H), 7.35 (s, 1H), 7.03 (t, *J* = 1.2, 2H), 6.99 (d, *J* = 8.4 Hz, 2H), 6.49 (t, *J* = 2, 1H), 6.42 (d, *J* = 16.4 Hz, 1H), 4.07 (s, 3H), 3.87 (s, 6H), 3.82 (s, 3H).

**(*E*)-Methyl-3-(7-methoxy-2-(2-methoxyphenyl)benzofuran-5-yl)acrylate (20c).**

Yellow solid 0.20g, 53.8% yield. ^1^H-NMR (CDCl_3_, 400 MHz) *δ* 8.10 (dd, *J* = 2.0 Hz, 8.0 Hz, 1H), 7.77 (d, *J* = 15.6 Hz, 1H), 7.32-7.36 (m, 3H), 7.05-7.09 (m, 1H), 7.01 (d, *J* = 8.0, 1H), 6.97 (d, *J* = 1.6 Hz, 1H), 6.41 (d, *J* = 16.0 Hz, 1H), 4.07 (s, 3H), 4.01 (s, 3H), 3.82 (s, 3H).

**(*E*)-Methyl-3-(7-methoxy-2-(3-methoxyphenyl)benzofuran-5-yl)acrylate (20d).**

Yellow solid 0.22g, 57.6% yield. ^1^H-NMR (CDCl_3_, 400 MHz) *δ* 7.77 (d, *J* = 16.0 Hz, 1H), 7.47 (d, *J* = 7.6 Hz, 1H), 7.41 (d, *J* = 2.4 Hz, 1H), 7.34-7.38 (m, 2H), 7.01 (s, 1H), 6.98 (d, *J* = 1.2 Hz, 1H), 6.92 (dd, *J* = 1.4 Hz, 8.2 Hz, 1H),6.42 (d, *J* = 16.0 Hz, 1H), 4.08 (s, 3H), 3.89 (s, 3H ), 3.82 (s, 3H).

**(*E*)-Methyl 3-(7-methoxy-2-(4-methoxyphenyl)benzofuran-5-yl) acrylate (20e).**

Yellow solid 0.28g, 60.7% yield. ^1^H-NMR (CDCl_3,_ 400 MHz) *δ* 7.82 (t, *J* = 2.4 Hz, 1H), 7.80 (t, *J* = 2.4 Hz, 1H), 7.76 (d, *J* = 15.6 Hz, 1H), 7.32 (s, 1H), 6.98 (t, *J* = 2.4 Hz, 1H) , 6.96 (t, *J* = 2.2 Hz, 2H), 6.76 (s, 1H), 6.41 (d, *J* = 15.6 Hz, 1H), 4.07 (s, 3H), 3.86 (s, 3H), 3.82 (s, 3H).

**(*E*)-Methyl-3-(2-(3,5-difluorophenyl)-7-methoxybenzofuran-5-yl)acrylate (20f).**

Yellow solid 0.03g, 28.9% yield. ^1^H-NMR (CDCl_3_, 400 MHz) *δ* 7.77 (d, *J* = 16.0 Hz, 1H), 7.36-7.39 (m, 3H), 7.05 (s, 1H), 7.01 (d, *J* = 1.0 Hz, 1H), 6.81(t, *J* = 2.0 Hz, 8.5Hz 1H), 6.43 (d, *J* = 16.0 Hz, 1H), 4.08 (s, 3H), 3.83 (s, 3H).

**(*E*)-Methyl 3-(2-(3,4-difluorophenyl)-7-methoxybenzofuran-5-yl)acrylate (20g).**

Yellow solid 0.18g, 78.9% yield. ^1^H-NMR (CDCl_3_, 400 MHz) *δ* 7.77 (d, *J* = 16.0 Hz, 1H), 7.66-7.71 (m, 1H), 7.59-7.62 (m, 1H), 7.35 (s, 1H), 7.21-7.27 (m, 1H), 7.00 (s, 1H), 6.97 (s, 1H), 6.43 (d, *J* = 16.0 Hz, 1H), 4.07 (s, 3H), 3.83 (s, 3H).

**(*E*)-Methyl 3-(2-(2,4-difluorophenyl)-7-methoxybenzofuran-5-yl)acrylate (20h).**

Yellow solid 0.19g, 75.9% yield. ^1^H-NMR (CDCl_3_, 400 MHz) *δ* 8.03-8.09 (m, 1H), 7.77 (d, *J* = 16.4 Hz, 1H), 7.38 (s, 1H), 7.16 (d, *J* = 3.6 Hz, 1H), 6.92-7.02 (m, 3H), 6.43 (d, *J* = 16.0 Hz, 1H), 4.07 (s, 3H), 3.83 (s, 3H).

**(*E*)-Methyl-3-(2-(2-fluorophenyl)-7-methoxybenzofuran-5-yl)acrylate (20i).**

Brown solid (0.44 g, 81.2 % yield). ^1^H NMR (CDCl_3_, 400 MHz) *δ* 8.08 (dt, *J* = 1.5 Hz *J* = 7.6 Hz, 1H), 7.78 (d, *J* = 16.0 Hz, 1H), 7.38 (s, 1H), 7.32-7.37 (m, 1H), 7.28 (s, 1H), 7.23 (d, *J* = 3.2 Hz, 1H), 7.21-7.16 (m, 1H), 7.0 (s, 1H), 6.43 (d, *J* = 16.0 Hz, 1H), 4.08 (s, 3H), 3.83 (s, 3H).

**(*E*)-Methyl-3-(2-(3-fluorophenyl)-7-methoxybenzofuran-5-yl)acrylate (20j).**

Pale yellow solid 0.34g, 88.7% yield. ^1^H-NMR (CDCl_3_, 400 MHz) *δ* 7.77 (d, *J* = 16.0 Hz, 1H ), 7.65 (dt, *J* = 1.1 Hz, 7.9 Hz, 1H), 7.58 (dt, *J* = 2.2 Hz, 9.6 Hz, 1H), 7.41 (dt, *J* = 5.6 Hz, 9.6 Hz, 1H), 7.36 (d, *J* = 1.6 Hz, 1H), 7.08 (dt, *J* = 5.2 Hz, 8.8 Hz, 1H), 7.03 (s, 1H ), 7.00 (d, *J* = 1.2 Hz, 1H), 6.42 (d, *J* = 16.0 Hz, 1H ), 4.08 (3H, s, CH_3_), 3.82 (3H, s, CH_3_).

**(*E*)-Methyl-3-(2-(4-fluorophenyl)-7-methoxybenzofuran-5-yl)acrylate (20k).**

Yellow solid 0.20g, 81.6% yield. ^1^H-NMR (CDCl_3_, 400 MHz) *δ* 7.86 (dd, *J* = 8.6 Hz *J* = 5.4 Hz, 2H), 7.77 (d, *J* = 15.6 Hz, 1H), 7.34 (s, 1H), 7.14 (t, *J* = 8.6 Hz, 2H), 6.98 (s, 1H), 6.95 (s, 1H), 6.42 (d, *J* = 15.6 Hz, 1H), 4.07 (s, 3H), 3.82 (s, 3H).

**(*E*)-Methyl-3-(7-methoxy-2-(2-(trifluoromethyl)phenyl)benzofuran-5-yl)acrylate (20l).**

Brown solid (0.31 g, 84.9 % yield). ^1^H NMR (CDCl_3_, 400 MHz) *δ* 7.90 (d, *J* = 7.6 Hz, 1H), 7.81 (d, *J* = 7.2 Hz, 1H), 7.76 (s, 1H), 7.64 (t, *J* = 8.0 Hz, 1H), 7.53 (t, *J* = 7.6 Hz, 1H), 7.40 (s, 1H), 7.26 (s, 1H), 7.03 (d, *J* = 6Hz, 2H), 6.43 (d, *J* = 15.6 Hz, 1H), 4.07 (s, 3H), 3.83 (s, 3H).

**(*E*)-Methyl-3-(7-methoxy-2-(3-(trifluoromethyl)phenyl)benzofuran-5-yl)acrylate (20m).**

Yellow solid 0.24g, 86.3% yield. ^1^H-NMR (CDCl_3_, 400 MHz) *δ* 8.12 (s, 1H ), 8.05 (d, *J* = 7.6 Hz, 1H), 7.77 (d, *J* = 15.6 Hz, 1H), 7.59 (t, *J* = 7.6 Hz, 2H), 7.37 (d, *J* = 1.2 Hz, 1H), 7.11 (s, 1H), 7.01 (d, *J* = 1.2 Hz,1H) ,6.43 (d, *J* = 15.6 Hz, 1H), 4.08 (s, 3H), 3.83 (s, 3H).

**(*E*)-Methyl-3-(7-methoxy-2-(4-(trifluoromethyl)phenyl)-1H-inden-5-yl)acrylate (20n).**

Yellow solid 0.15g, 87.2% yield. ^1^H-NMR (CDCl_3_, 400 MHz) *δ* 7.98 (d, *J* = 8.0 Hz, 2H), 7.77 (d, *J* = 16.0 Hz, 1H), 7.70 (t, *J* = 8.0 Hz, 2H), 7.38 (d, *J* = 1.6 Hz, 1H), 7.13 (s, 1H), 7.02 (d, *J* = 1.2 Hz, 1H) ,6.43 (d, *J* = 15.6 Hz, 1H), 4.08 (s,3H), 3.82 (s, 3H).

**(*E*)-Methyl-3-(2-(4-(dimethylamino)phenyl)-7-methoxybenzofuran-5-yl)acrylate (20o).**

Yellow solid 0.22g, 79.2% yield. ^1^H-NMR (CDCl_3_, 400 MHz) *δ* 7.7 (t, *J* = 7.4 Hz, 3H), 7.30 (s, 1H), 6.93 (s, 1H), 6.79 (s, 3H), 6.40 (d, *J* = 16.4 Hz, 1H), 4.07 (s, 3H), 3.82 (s, 3H), 3.03 (s, 6H).

**(*E*)-Methyl 3-(2-(4-(hydroxymethyl)phenyl)-7-methoxybenzofuran-5-yl)acrylate (20p).**

Yellow solid 0.21g, 75.2% yield. ^1^H-NMR (CDCl_3_, 400 MHz) *δ* 7.88 (d, *J* = 8.0 Hz, 1H), 7.77 (d, *J* = 16.0 Hz, 1H), 7.34-7.40 (m, 3H), 7.16 (t, *J* = 7.2 Hz, 1H); 7.07 (d, *J* = 8.8 Hz, 4H), 6.97 (d, *J* = 1.2 Hz, 1H), 6.93 (s, 1H), 6.42 (d, *J* = 16.0 Hz, 1H), 4.07 (s, 3H), 3.82 (s, 3H).

**(*E*)-Methyl 3-(7-methoxy-2-(4-phenoxyphenyl)benzofuran-5-yl)acrylate (20q).**

Yellow solid 0.24g, 75.2% yield. ^1^H-NMR (CDCl_3_, 400 MHz) *δ* 7.84 (q, *J* = 2.0 Hz, 1H), 7.77 (d, *J* = 16.0 Hz, 1H), 7.45 (d, *J* = 8.4 Hz, 2H), 7.35 (s, 1H), 7.01 (s,1H), 6.98 (s,1H), 6.42 (d, *J* = 16.0 Hz, 1H), 4.76 (d, *J* = 10.0 Hz, 2H), 4.08 (s, 3H), 3.82 (s, 3H), 1.74 (t, *J* = 6.0 Hz, 1H).

**General procedure of hydrogenation reaction for synthesis of 21a-q.**

To a solution of acrylate (1 equiv.) in THF (10 volume) was added Pd/C (0.1 equiv., 10 wt%), followes by one or two drops of CH_3_COOH and bubbled with hydrogen at room temperature for about 30 minutes under stirring. After completion of the reaction, the reaction mixture was concentrated under reduced pressure. The resulting residue was purified by silica gel column chromatography to give white solid 21a-q.

**Methyl 3-(7-methoxy-2-phenylbenzofuran-5-yl)propanoate (21a).**

White solid 0.13g, 93.3% yield. ^1^H-NMR (CDCl_3_, 400 MHz) *δ* 7.87 (d, *J* = 7.6 Hz, 2H), 7.87 (t, *J* = 7.6 Hz, 2H), 7.35 (d, *J* = 7.6 Hz, 1H), 7.00 (s, 1H), 6.96 (s, 1H), 6.65 (s, 1H), 4.04 (s, 3H), 3.68 (s, 3H), 3.02 (t, *J* = 7.8 Hz, 2H), 2.69 (t, *J* = 7.8 Hz, 2H).

**Methyl-3-(2-(3,5-dimethoxyphenyl)-7-methoxybenzofuran-5-yl)propanoate (21b).**

Yield white solid 0.15g, 87.2% yield. ^1^H-NMR (CDCl_3,_ 400 MHz) *δ* 7.01 (q, *J* = 3.3 Hz, 3H), 6.94 (s, 1H), 6.65 (d, *J* = 1.2 Hz, 1H), 6.46 (t, *J* = 2.4, 1H), 4.03 (s, 3H), 3.86 (s, 6H), 3.68 (s, 3H), 3.02 (t, *J* = 8.0, 2H), 2.68 (t, *J* = 7.8, 2H).

**Methyl-3-(7-methoxy-2-(2-methoxyphenyl)benzofuran-5-yl)propanoate (21c).**

White solid 0.11g, 86.6% yield. ^1^H-NMR (CDCl_3_, 400 MHz) *δ* 8.10 (dd, *J* = 1.6, 8.0 Hz, 1H), 7.29-7.34 (m, 1H), 7.28 (s, 1H), 7.06 (t, *J* = 7.6 Hz, 1H), 7.00 (d, *J* = 8.4 Hz, 2H), 6.64 (d, *J* = 1.2 Hz, 1H), 4.04 (s, 3H), 4.00 (s, 3H), 3.68 (s, 3H), 3.02 (t, *J* = 7.8 Hz, 2H), 2.69 (t, *J* = 7.8 Hz, 2H).

**Methyl-3-(7-methoxy-2-(3-methoxyphenyl)benzofuran-5-yl)propanoate (21d).**

Yellow solid 0.12 mg, 89.5% yield. ^1^H-NMR (CDCl_3_, 400 MHz) *δ* 7.46 (d, *J* = 7.6 Hz, 2H), 7.40 (s, 1H), 7.34 (t, *J* = 7.8 Hz, 1H), 7.00 (s, 1H), 6.94 (s, 1H) 6.90 (dd, *J* = 2.2 Hz, 7.8 Hz, 1H), 6.65 (s, 1H), 4.03 (s, 3H), 3.88 (s, 3H), 3.68(s, 3H), 3.02 (t, *J* = 7.8 Hz, 2H), 2.68 (t, *J* = 7.6Hz, 2H).

**Methyl-3-(7-methoxy-2-(4-methoxyphenyl)benzofuran-5-yl) propanoate (21e).**

Yellow solid 0.13g, 87.9% yield. ^1^H-NMR (CDCl_3_, 400 MHz) *δ* 7.80 (q, *J* = 2.8 Hz, 2H), 6.95-6.98 (m, 3H), 6.81 (s, 1H), 6.62 (d, *J* = 1.2 Hz, 1H), 4.03 (s, 3H), 3.85 (s, 3H), 3.68 (s, 3H), 3.02 (t, *J* = 8.0 Hz, 2H), 2.68 (t, *J* = 7.8 Hz, 2H).

**Methyl-3-(2-(3,5-difluorophenyl)-7-methoxybenzofuran-5-yl)propanoate (21f).**

Yellow solid 0.11g, 89.5% yield. ^1^H-NMR (CDCl_3_, 400 MHz) *δ* 7.35-7.38 (m, 2H), 7.01 (d, *J* = 0.8 Hz, 1H), 6.98 (s, 1H), 6.75-6.80 (m, 1H,), 6.68 (d, *J* = 1.6 Hz, 1H), 4.03 (s, 3H), 3.68 (s, 3H), 3.03 (t, J = 7.8 Hz, 2H). 2.68 (t, J = 7.8 Hz, 2H).

**Methyl 3-(2-(3,4-difluorophenyl)-7-methoxybenzofuran-5-yl)propanoate (21g).**

Yellow solid 0.15g, 90.5% yield. ^1^H-NMR (CDCl_3_, 400 MHz) *δ* 7.64-7.70 (m, 1H), 7.57-7.59 (m, 1H), 7.20 (t, J = 7.8 Hz, 1H), 7.01 (d, *J* = 0.8 Hz, 1H), 7.00 (s, 1H), 6.90 (s, 1H), 6.70 (s, 1H), 4.03 (s, 3H), 3.68 (s, 3H), 3.03 (t, J = 7.8 Hz, 2H). 2.68 (t, J = 7.8 Hz, 2H).

**Methyl 3-(2-(2,4-difluorophenyl)-7-methoxybenzofuran-5-yl)propanoate (21h).**

Yellow solid 0.26g, 95.5% yield. ^1^H-NMR (CDCl_3_, 400 MHz) *δ* 8.02-8.08 (m, 1H), 7.10 (d, *J* = 3.6 Hz, 1H), 7.02 (s, 1H), 6.90-7.00 (m, 2H), 6.67 (s, 1H), 4.03 (s, 3H), 3.68 (s, 3H), 3.03 (t, J = 7.8 Hz, 2H). 2.69 (t, J = 7.8 Hz, 2H).

**Methyl-3-(2-(2-fluorophenyl)-7-methoxybenzofuran-5-yl)propanoate (21i).**

White solid 0.07 g, 56.1 % yield. ^1^H-NMR (CDCl_3_, 400 MHz) *δ*  8.09-8.05 (m, 1H), 7.33-7.28 (m, 1H), 7.23 (d, *J* = 7.2 Hz, 1H), 7.16 (dd, *J* = 8.2 Hz, 11.4 Hz, 2H), 7.03 (s, 1H), 6.67 (s, 1H), 4.04 (s, 3H), 3.69 (s, 3H), 3.03 (t, *J* = 7.8 Hz, 2H), 2.68 (t, *J* = 7.8 Hz, 2H).

**Methyl-3-(2-(3-fluorophenyl)-7-methoxybenzofuran-5-yl)propanoate (21j).**

White solid 0.16g, 80.3% yield. ^1^H-NMR (CDCl_3_, 400 MHz) *δ* 7.64 (d, *J* = 8.0 Hz, 1H), 7.57 (dt, *J* = 2.0 Hz, 9.9 Hz, 1H), 7.39 (dt, *J* = 5.9 Hz, 8.0 Hz, 1H), 7.04 (dd, *J* = 2.2 Hz, 8.2 Hz, 1H), 7.01 (d, *J* = 0.8 Hz, 1H), 6.97 (s, 1H), 6.67 (d, *J* = 1.2 Hz, 1H) 4.03 (s, 3H), 3.68 (s, 3H), 3.03 (t, *J* = 7.8 Hz, 2H), 2.69 (t, *J* = 7.8 Hz, 2H).

**Methyl-3-(2-(4-fluorophenyl)-7-methoxybenzofuran-5-yl)propanoate (21k).**

Yellow solid 0.08g, 80.0% yield. ^1^H-NMR (CDCl_3_, 400 MHz) *δ* 7.84 (dd, *J* = 5.4 Hz *J* = 8.6 Hz, 2H), 7.12 (t, *J* = 8.6 Hz, 2H), 7.00 (s, 1H), 6.88 (s, 1H), 6.65 (s, 1H), 4.03 (s, 3H), 3.68 (s, 3H) 3.02 (t, *J* = 7.8 Hz, 2H), 2.69 (t, *J* = 7.8 Hz, 2H).

**Methyl-3-(7-methoxy-2-(2-(trifluoromethyl)phenyl)benzofuran-5-yl)propanoate (21l).**

Yellow liquid 0.06 g, 58.7 % yield. ^1^H-NMR (CDCl_3_, 400 MHz) *δ* 7.89 (d, *J* = 7.6 Hz, 1H), 7.79 (d, *J* = 8.0 Hz, 1H), 7.62 (t, *J* = 7.6 Hz, 1H), 7.50 (t, *J* = 7.8 Hz, 1H), 7.05 (s, 1H), 6.97 (s, 1H), 6.70 (s, 1H), 4.03 (s, 3H), 3.69 (s, 3H), 3.04 (t, *J* = 7.8 Hz, 2H), 2.70 (t, *J* = 7.6 Hz, 2H).

**Methyl-3-(7-methoxy-2-(3-(trifluoromethyl)phenyl)benzofuran-5-yl)propanoate (21m).**

White solid 0.09g, 68.2% yield. ^1^H-NMR (CDCl_3_, 400 MHz) *δ* 8.10 (s,1H), 8.03 (d, *J* = 7.2 Hz, 1H), 7.57 (t, *J* = 5.4 Hz, 1H), 7.04 (s, 1H), 7.02 (d, *J* = 0.08 Hz, 1H), 6.68 (s, 1H), 4.04 (s, 3H), 3.69 (s, 3H), 3.03 (t, *J* = 7.8 Hz, 2H), 2.69 (t, *J* = 7.8 Hz, 2H).

**Methyl-3-(7-methoxy-2-(4-(trifluoromethyl)phenyl)benzofuran-5-yl)propanoate (21n).**

White solid 0.09g, 68.2% yield. ^1^H-NMR (CDCl_3_, 400 MHz) *δ* 7.97 (d, *J* = 8.4 Hz, 2H), 7.68 (d, *J* = 8.4 Hz, 2H), 7.07 (s, 1H), 7.03 (d, *J* = 1.2 Hz, 1H), 6.69 (d, *J* = 1.6 Hz, 1H), 4.04 (s, 3H), 3.68 (s, 3H), 3.03 (t, *J* = 7.8 Hz, 2H), 2.69 (t, *J* = 7.8 Hz, 2H).

**Methyl-3-(2-(4-(dimethylamino)phenyl)-7-methoxybenzofuran-5-yl)propanoate (21o).**

White solid 0.10g, 67.6% yield. ^1^H-NMR (CDCl_3_, 400 MHz) *δ* 7.74 (d, *J* = 9.2 Hz, 2H), 6.94 (d, *J* = 2.0 Hz, 1H), 6.75 (d, *J* = 8.8 Hz 2H), 6.72 (s, 1H), 6.59 (d, *J* = 2.0 Hz, 1H), 4.03 (s, 3H), 3.68 (s, 3H), 3.01 (t, *J* = 7.8 Hz, 2H), 3.01 (s, 6H), 2.67 (t, *J* = 7.8 Hz, 2H).

**Methyl 3-(2-(4-(hydroxymethyl)phenyl)-7-methoxybenzofuran-5-yl)propanoate (21p).**

White solid 0.15g, 73.6% yield. ^1^H-NMR (CDCl_3_, 400 MHz) *δ* 7.87 (d, *J* = 8.4 Hz, 2H), 7.44 (d, *J* = 8.0 Hz, 1H), 7.00 (s, 1H), 6.95 (s, 1H), 6.65 (s, 1H), 4.74 (d, *J* = 6.0 Hz, 2H), 4.04 (s, 3H), 3.68 (s, 3H), 3.03 (t, *J* = 7.8 Hz, 2H), 2.69 (t, *J* = 7.8 Hz, 2H), 1.70 (t, *J* = 6.0 Hz, 1H).

**Methyl 3-(7-methoxy-2-(4-phenoxyphenyl)benzofuran-5-yl)propanoate (21q).**

White solid 0.17g, 90.6% yield. ^1^H-NMR (CDCl_3_, 400 MHz) *δ* 7.83 (q, *J* = 2.2 Hz, 2H), 7.35-7.39 (m, 2H), 7.12-7.16 (m, 1H), 7.04-7.07 (m, 4H), 6.99 (d, *J* = 1.2 Hz, 1H), 6.86 (s, 1H), 6.64 (d, *J* = 1.6 Hz, 2H), 4.03 (s, 3H), 3.68 (s, 3H), 3.02 (t, *J* = 7.8 Hz, 2H), 2.68 (t, *J* = 7.8 Hz, 2H).

**General procedure of reduction ester for synthesis of (22a-q).**

To a solution of ester (1.0 equiv.) in THF (10 voulme) was added LiAlH_4_ in THF (2.0 M, 1.0 equiv) dropwise at 0 ˚C, then the mixture was stirred at room temperature until the reaction was complete as judged by TLC. The reaction mixture was then acidified with 10% HCl and then partitioned between EtOAc and brine. The organic layer was separated, dried over anhydrous MgSO_4_, filtered, and concentrated under *vacuo*. The resulting residue was purified by silica gel column chromatography to obtain white solid **22a-q**.

**3-(7-Methoxy-2-phenylbenzofuran-5-yl)propan-1-ol (22a).**

White solid 0.06g, 78.5% yield. ^1^H-NMR (CDCl_3_, 400 MHz) *δ* 7.89 (d, *J* = 0.8 Hz, 1H), 7.87 (s, 1H), 7.43 (t, *J* = 7.6 Hz, 2H), 7.35 (d, *J* = 7.6 Hz, 1H), 7.01 (s, 1H), 6.96 (s, 1H), 6.66 (s, 1H), 4.04 (s, 3H), 3.72 (q, *J* = 9.2 Hz, 2H), 2.79 (t, *J* = 7.6 Hz, 2H), 1.92-1.99 (m, 2H), 1.29(t, *J* = 5.2 Hz, 1H); ^13^C-NMR (CDCl_3,_ 100 MHz) *δ* 156.2, 144.9, 142.7, 137.5, 130.9, 130.4, 128.7, 128.5, 125.0, 112.5, 107.5, 101.5, 62.3, 56.2, 56.1, 34.7, 32.4; HRMS [M + H]^+^ calcd [C_18_H_19_O_3_], 283.1334; found, 2831336 ; Purity 99.97% (as determined by RP-HPLC, method A, *t*_R_ = 16.60 min).

**3-(2-(3,5-Dimethoxyphenyl)-7-methoxybenzofuran-5-yl)propan-1-ol (22b).**

White solid 0.04g, 90.1% yield. ^1^H-NMR (CDCl_3,_ 400 MHz) *δ* 7.02 (d, *J* = 2.0 Hz, 1H), 7.00 (d, *J* = 1.2 Hz, 1H), 6.94 (s, 1H), 6.66 (d, *J* = 0.8 Hz, 1H), 6.46 (t, *J* = 6.4 Hz, 1H), 4.04 (s, 3H), 3.87 (s, 6H), 3.72 (q, *J* = 5.7 Hz, 2H), 2.79 (t, *J* = 7.8 Hz, 2H), 1.91-1.98 (m, 2H), 1.31 (t, *J* = 5.0 Hz, 1H); ^13^C-NMR (CDCl_3,_ 100 MHz) *δ* 161.0, 156.0, 144.9, 142.7, 132.1, 130.8, 112.5, 107.7, 103.1, 102.0, 101.0, 62.3, 56.1, 55.5, 34.6, 32.4; HRMS [M + H]^+^ calcd [C_20_H_22_O_5_], 343.1545; found, 343.1548; Purity 99.98% (as determined by RP-HPLC, method A, *t*_R_ = 17.02 min).

**3-(7-Methoxy-2-(2-methoxyphenyl)benzofuran-5-yl)propan-1-ol (22c).**

White solid 0.07g, 85% yield. ^1^H-NMR (CDCl_3_, 400 MHz) *δ* 8.11 (dd, *J* = 1.6 Hz, 8.0 Hz, 1H), 7.29-7.31 (m, 1H), 7.28 (s, 1H), 7.06 (t, *J* = 7.6 Hz, 1H), 7.01 (d, *J* = 0.8, 1H), 7.00 (d, *J* = 8.4, 1H), 6.64 (s, 1H), 4.04 (s, 3H), 3.99 (s, 3H), 3.71 (t, *J* = 6.4 Hz,2H), 2.78 (t, *J* = 7.6 Hz, 2H), 1.91-1.97 (m, 2H), 0.885 (t, *J* = 5.0 Hz, 1H); ^13^C-NMR (CDCl_3,_ 100 MHz) *δ* 156.3, 152.4, 144.7, 141.7, 137.1, 131.5, 129.1, 127.2, 120.7, 119.2, 112.6, 110.9, 107.5, 106.4, 62.3, 56.1, 55.4, 34.7, 32.4; HRMS [M + H]^+^ calcd [C_19_H_20_O_4_], 313.1440; found, 313.1437; Purity 99.99% (as determined by RP-HPLC, method A, *t*_R_ = 17.32 min).

**3-(7-Methoxy-2-(3-methoxyphenyl)benzofuran-5-yl)propan-1-ol (22d).**

White solid 0.07g, 75.8% yield. ^1^H-NMR (CDCl_3_, 400 MHz) *δ* 7.47 (d, *J* = 8.0 Hz, 1H), 7.40 (d, *J* = 2.4 Hz, 1H), 7.34 (t, *J* = 8.0 Hz, 1H), 7.00 (s, 1H), 6.95 (s, 1H), 6.90 (dd, *J* = 1.8 Hz, 7.8 Hz, 1H), 6.66 (s, 1H), 4.04 (s, 3H), 3.89 (s, 3H), 3.72 (q, *J* = 6.0 Hz, 2H), 2.79 (t, *J* = 8.0 Hz, 2H), 1.93-1.99 (m, 2H), 1.27 (t, *J* = 5.4 Hz, 1H); ^13^C-NMR (CDCl_3,_ 100 MHz) *δ* 159.8, 156.0, 144.9, 142.7, 137.5, 131.6, 130.8, 129.7, 117.6, 114.4, 112.5, 110.2, 107.7, 101.8, 62.3, 56.1, 55.4, 34.6, 32.4; HRMS [M + H]^+^ calcd [C_19_H_21_O_4_], 313.1440; found, 313.1439; Purity 99.99% (as determined by RP-HPLC, method A, *t*_R_ = 17.10 min).

**3-(7-Methoxy-2-(4-methoxyphenyl)benzofuran-5-yl)propan-1-ol(22e).**

White solid 0.08g, 73.8% yield. ^1^H-NMR (CDCl_3_, 400 MHz) *δ* 7.80 (t, J = 5.6 Hz, 2H), 6.99 (d, *J* = 6.0 Hz, 2H), 6.95 (s, 1H), 6.63 (s, 1H), 4.04 (s, 3H), 3.85 (s, 3H), 3.72 (q, *J* = 6.0 Hz, 2H), 2.78 (t, *J* = 7.6 Hz, 2H), 1.91-1.98 (m, 2H), 1.26 (t, *J* = 6.0 Hz, 1H); ^13^C-NMR (CDCl_3,_ 100 MHz) *δ* 159.9, 156.4, 144.7, 142.4, 137.4, 131.2, 126.4, 123.2, 114.1, 112.2, 107.2, 99.8, 62.3, 56.1, 55.3, 34.7, 32.4; HRMS [M + H]^+^ calcd [C_19_H_20_O_4_], 313.1440; found, 313.1433; Purity 99.99% (as determined by RP-HPLC, method A, *t*_R_ = 16.84 min).

**3-[2-(3,5-Difluorophenyl)-7-methoxybenzofuran-5-yl]propan-1-ol (22f).**

White solid 0.05g, 28.5% yield. ^1^H-NMR (CDCl_3_, 400 MHz) *δ* 7.35-7.40 (m, 1H), 7.02 (s, 1H), 6.98 (s, 1H), 6.74-6.80 (m, 1H), 6.69 (s, 1H), 4.06 (s, 3H), 3.72 (q, *J* = 6.0 Hz, 2H), 2.79 (t, *J* = 7.8 Hz, 2H), 1.87-2.04 (m, 2H), 1.26 (t, *J* = 5.0 Hz, 1H); ^13^C-NMR (CDCl_3,_ 100 MHz) *δ* 164.8, 162.2, 145.0, 142.9, 138.0, 133.4, 130.3, 112.7, 108.3, 107.8, 103.5, 62.2, 56.1, 34.6, 32.4; HRMS [M + H]^+^ calcd [C_18_H_16_O_3_F_2_], 319.1146; found, 319.1146; Purity 99.98% (as determined by RP-HPLC, method A, *t*_R_ = 19.00 min).

**3-(2-(3,4-Difluorophenyl)-7-methoxybenzofuran-5-yl)propan-1-ol (22g).**

White solid 0.08g, 75.9% yield. ^1^H-NMR (CDCl_3_, 400 MHz) *δ* 7.65-7.70 (m, 1H), 7.57-7.60 (m, 1H), 7.22 (q, *J* = 8.4 Hz, 1H), 7.00 (s, 1H), 6.90 (s, 1H), 6.67 (s, 1H), 4.03 (s, 3H), 3.72 (q, *J* = 6.0 Hz, 2H), 2.79 (t, *J* = 7.8 Hz, 2H), 1.91-1.98 (m, 2H), 1.30 (t, *J* = 5.2 Hz, 1H); ^13^C-NMR (CDCl_3,_ 100 MHz) *δ* 154.1, 154.0, 151.9, 151.8, 151.7, 151.6, 149.4, 149.3, 149.2, 149.0, 144.9, 142.9, 137.9, 130.6, 127.6, 127.6, 127.6, 127.5, 121.2, 121.2, 121.1, 121.1, 114.1, 114.0, 112.6, 107.9, 102.2, 62.3, 56.1, 34.6, 32.4 ; HRMS [M + H]^+^ calcd [C_18_H_17_O_3_F_2_], 319.1146; found, 319.1159; Purity 99.99% (as determined by RP-HPLC, method A, *t*_R_ = 18.34 min).

**3-(2-(2,4-Difluorophenyl)-7-methoxybenzofuran-5-yl)propan-1-ol (22h).**

White solid 0.07g, 75.4% yield. ^1^H-NMR (CDCl_3_, 400 MHz) *δ* 8.02-8.08 (m, 1H), 7.10 (d, *J* = 4.0 Hz, 1H), 7.03 (s, 1H), 6.90-7.01 (m, 2H), 6.68 (s, 1H), 4.04 (s, 3H), 3.72 (q, *J* = 5.2 Hz, 2H), 2.79 (t, *J* = 7.6 Hz, 2H), 1.92-1.99 (m, 2H), 1.29 (t, *J* = 5.2 Hz, 1H); ^13^C-NMR (CDCl_3,_ 100 MHz) *δ* 144.9, 142.1, 137.8, 130.9, 128.3, 128.2, 128.1, 112.8, 111.8, 111.6, 107.7, 106.1, 106.0, 104.8, 104.6, 104.3, 62.3, 56.1, 34.8, 32.4; HRMS [M + H]^+^ calcd [C_18_H_16_O_3_F_2_], 319.1146; found, 319.1162; Purity 99.98% (as determined by RP-HPLC, method A, *t*_R_ = 18.54 min).

**3-(2-(2-Fluorophenyl)-7-methoxybenzofuran-5-yl)propan-1-ol (22i).**

Yelow solid (0.036 g, 80 % yield). ^1^H NMR (CDCl_3_, 400 MHz) *δ* = 8.09-8.05 (m, 1H), 7.34-7.28 (m, 1H), 7.23 (dd, *J* = 1.2 Hz, 7.6 Hz, 1H), 7.17 (dd, *J* = 8.2 Hz, 11.4 Hz, 2H), 7.04 (s, 1H), 6.68 (d, *J* = 0.8 Hz, 1H), 4.05 (s, 3H), 3.72 (t, *J* = 5.2 Hz, 2H), 2.80 (t, *J* = 7.8 Hz, 2H), 1.99-1.92 (m, 2H); ^13^C-NMR (CDCl_3,_ 100 MHz) *δ* 160.7, 158.2, 150.1, 150.0, 144.8, 142.1, 137.6, 131.0, 129.5, 129.4, 124.4, 124.3, 118.7, 118.6, 116.1, 115.8, 112.8, 107.9, 106.8, 106.7, 62.3, 56.1, 34.7, 32.4, HRMS [M + H]^+^ calcd [C_18_H_17_O_3_F], 301.1240; found, 301.1245; Purity 99.99% (as determined by RP-HPLC, method A, *t*_R_ = 17.70 min).

**3-(2-(3-Fluorophenyl)-7-methoxybenzofuran-5-yl)propan-1-ol (22j).**

White solid 115mg , 95.8% yield .^1^H-NMR (CDCl_3_, 400 MHz) *δ* 7.64 (d, *J* = 8.0 Hz, 1H) 7.57 (d, *J* = 9.6 Hz, 1H), 7.39, (q, *J* = 7.3 Hz, 1H), 7.01-7.05 (m, 2H), 6.97 (s, 1H), 4.04 (s, 3H), 3.72 (q, *J* = 6.0 Hz, 2H), 2.79 (t, *J* = 7.8 Hz, 2H), 1.92-1.99 (m, 2H), 1.27 (q, *J* = 5.2 Hz, 1H); ^13^C-NMR (CDCl_3,_ 100 MHz) *δ* 164.3, 161.8, 154.8, 144.9, 142.8, 137.7, 132.5, 130.5, 120.6, 115.2, 112.6, 111.9, 107.9, 102.5, 62.2, 56.1, 34.6, 32.4; HRMS [M + H]^+^ calcd [C_18_H_20_O_3_F], 301.1240; found, 301.1241; Purity 99.99% (as determined by RP-HPLC, method A, *t*_R_ = 17.77 min).

**3-(2-(4-Fluorophenyl)-7-methoxybenzofuran-5-yl)propan-1-ol (22k).**

Yellow solid 0.05g, 69.4% yield. ^1^H-NMR (CDCl_3_, 400 MHz) *δ* 7.82-7.86 (m, 2H) 7.12 (t, *J* = 8.8 Hz, 2H), 7.00 (s, 1H), 6.88 (s, 1H), 6.65 (s, 1H), 4.04 (s, 3H), 3.71 (t, *J* = 6.4 Hz, 2H), 2.79 (t, *J* = 7.8 Hz, 2H), 1.91-1.98 (m, 2H), 1.27(d, *J* = 8.8 Hz, 1H); ^13^C-NMR (CDCl_3,_ 100 MHz) *δ* 164.1, 161.6, 155.4, 144.9, 137.7, 130.9, 126.9, 126.8, 126.7, 126.7, 115.9, 115.7, 112.5, 107.6, 101.2, 62.3, 56.1, 34.7, 32.4; HRMS [M + H]^+^ calcd [C_18_H_17_O_3_F], 301.1240; found, 301.1248; Purity 99.99% (as determined by RP-HPLC, method A, *t*_R_ = 17.52 min).

**3-(7-Methoxy-2-(2-(trifluoromethyl)phenyl)benzofuran-5-yl)propan-1-ol (22l).**

Transparent liquid (0.04 g, 67.2 % yield). ^1^H NMR (CDCl_3_, 400 MHz) *δ* = 7.90 (d, *J* = 7.6 Hz, 1H), 7.79 (d, *J* = 8.0 Hz, 1H), 7.62 (t, *J* = 7.6 Hz, 1H), 7.50 (t, *J* = 7.8 Hz, 1H), 7.05 (s, 1H), 6.98 (s, 1H), 6.70 (d, *J* = 0.8 Hz, 1H), 4.04 (s, 3H), 3.73 (t, *J* = 6.4 Hz, 2H), 2.80 (t, *J* = 7.6 Hz, 2H), 2.00-1.93 (m, 1H); ^13^C-NMR (CDCl_3,_ 100 MHz) *δ* 152.6, 144.9, 143.0, 137.7, 131.7, 131.1, 130.5, 129.5, 129.4, 127.7, 128.1, 128.0, 127.8, 127.4, 127.1, 126.7, 126.6, 126.5, 126.5, 125.2, 122.5, 62.2, 56.3, 34.7, 32.4; HRMS [M + H]^+^ calcd [C_19_H_17_O_3_F_3_], 351.1208; found, 351.1209; Purity 99.98% (as determined by RP-HPLC, method A, *t*_R_ = 18.42 min).

**3-(7-Methoxy-2-(3-(trifluoromethyl)phenyl)benzofuran-5-yl)propan-1-ol (22m).**

White solid 0.05g, 79.4% yield. ^1^H-NMR (CDCl_3_, 400 MHz) *δ* 8.10 (s, 1H) 8.03 (d, *J* = 7.2 Hz, 1H), 7.52-7.59 (m, 2H), 7.04 (s, 1H),7.02 (d, *J* = 1.2 Hz, 1H), 6.69 (d, *J* = 1.2 Hz, 1H), 4.05 (s, 3H), 3.72 (d, *J* = 2.4 Hz, 2H), 2.80 (t, *J* = 7.6 Hz, 2H), 1.92-1.99 (m, 2H), 1.28(d, *J* = 29.2 Hz, 1H); ^13^C-NMR (CDCl_3,_ 100 MHz) *δ* 154.5, 145.0, 142.9, 137.9, 131.1, 130.5, 129.2, 1273.9, 124.9, 121.6, 112.6, 108.0, 102.8, 62.2, 56.1, 34.6, 32.4; HRMS [M + H]^+^ calcd [C_19_H_17_O_3_F_3_], 351.1208; found, 351.1214; Purity 99.97% (as determined by RP-HPLC, method A, *t*_R_ = 15.93 min).

**3-(7-methoxy-2-(4-(trifluoromethyl)phenyl)benzofuran-5-yl)propan-1-ol (22n).**

White solid 0.50g, 79.4% yield. ^1^H-NMR (CDCl_3_, 400 MHz) *δ* 7.97 (d, *J* = 8.8 Hz, 2H) 7.67 (d, *J* = 8.4 Hz, 2H), 7..06 (s, 1H), 7.03 (d, *J* = 0.8 Hz, 1H), 6.69 (d, *J* = 1.2 Hz, 1H), 4.04 (s, 3H), 3.72 (t, *J* = 6.2 Hz, 2H), 2.80 (t, *J* = 7.6Hz, 2H), 1.92-1.99 (m, 2H), 1.35(s, 1H); ^13^C-NMR (CDCl_3,_ 100 MHz) *δ* 154.5, 145.0, 143.1, 137.9, 133.6, 130.5, 125.7, 125.0, 122.7, 112.7, 108.1, 103.4, 62.2, 56.1, 34.6, 32.4; HRMS [M + H]^+^ calcd [C_19_H_17_F_3_O_3_], 351.1208; found, 351.1220; Purity 99.99% (as determined by RP-HPLC, method A, *t*_R_ = 20.44 min).

**3-(2-(4-(dimethylamino)phenyl)-7-methoxybenzofuran-5-yl)propan-1-ol (22o).**

White solid 0.06g, 78.6% yield. ^1^H-NMR (CDCl_3_, 400 MHz) *δ* 7.74 (q, *J* = 3.2 Hz, 2H), 6.95 (d, *J* = 1.2 Hz, 1H), 6.75 (d, *J* = 8.8 Hz, 2H), 6.72 (s, 1H), 6.59 (d, *J* = 1.6 Hz, 1H), 4.04 (s, 3H), 3.71 (q, *J* = 6.0 Hz, 2H), 3.01 (s, 6H), 2.77 (t, *J* = 7.6 Hz, 2H ), 1.91-1.98 (m, 2H ), 1.25(t, *J* = 5.4 Hz, 1H ); ^13^C-NMR (CDCl_3,_ 100 MHz) *δ* 157.4, 150.5, 144.6, 142.1, 137.1, 131.5, 126.2, 118.5, 112.0, 106.7, 98.2, 62.3, 56.1, 40.3, 34.7, 32.4; HRMS [M + H]^+^ calcd [C_20_H_23_NO_3_], 326.1756; found, 326.1753; Purity 99.97% (as determined by RP-HPLC, method A, *t*_R_ = 10.64 min).

**3-(2-(4-(Hydroxymethyl)phenyl)-7-methoxybenzofuran-5-yl)propan-1-ol (22p).**

White solid 0.05g, 64.6% yield. ^1^H-NMR (CDCl_3_, 400 MHz) *δ* 7.87 (d, *J* = 8.4 Hz, 2H), 7.44 (d, *J* = 8.0 Hz, 2H), 7.00 (s, 1H), 6.95 (s, 1H), 6.66 (s, 1H), 4.74 (d, *J* = 6.0 Hz, 1H), 4.04 (s, 3H), 3.72 (q, *J* = 6.2 Hz, 2H), 2.79 (t, *J* = 7.8 Hz, 2H), 1.93-1.99 (m, 2H), 1.07(t, *J* = 5.8 Hz, 1H), 1.30(d, *J* = 1.2 Hz, 1H); ^13^C-NMR (CDCl_3,_ 100 MHz) *δ* 156.0, 144.9, 142.7, 141.2, 137.6, 130.9, 129.8, 127.3, 125.2, 112.5, 107.6, 101.5, 65.1, 62.3, 56.2, 34.7, 32.4; HRMS [M + H]^+^ calcd [C_19_H_21_O_4_], 313.1440; found, 313.1443; Purity 99.97% (as determined by RP-HPLC, method A, *t*_R_ = 10.92 min).

**3-(7-Methoxy-2-(4-phenoxyphenyl)benzofuran-5-yl)propan-1-ol (22q)**

White solid 0.07g, 90.5% yield. ^1^H-NMR (CDCl_3_, 400 MHz) *δ* 7.81-7.85 (m, 2H), 7.35-7.39 (m, 2H), 7.12-7.16 (m, 1H), 7.03-7.07 (m, 4H), 7.00 (s, 2H), 6.87 (s, 1H), 6.65 (d, *J* = 2.0 Hz, 1H), 4.04 (s, 3H), 3.72 (q, *J* = 6.2 Hz, 2H), 2.79 (t, *J* = 7.8 Hz, 2H), 1.92-1.99 (m, 2H), 1.27(t, *J* = 5.4 Hz, 1H); ^13^C-NMR (CDCl_3,_ 100 MHz) *δ* 157.7, 156.7, 155.9, 144.9, 142.6, 137.6, 131.1, 129.9, 126.6, 125.5, 123.7, 119.2, 118.8, 112.4, 107.5, 100.7, 62.3, 56.2, 34.7, 32.4; HRMS [M + H]^+^ calcd [C_24_H_23_O_4_], 375.1596; found, 375.1594; Purity 99.98% (as determined by RP-HPLC, method A, *t*_R_ = 22.00 min).

**Scheme S1**

^a^**Reagents and conditions:** a) CBr_4_, PPh_3_, DCM; b) LDA, THF;c) 10% Pd/C, PPh_3_, CuI, Et_3_N, H_2_O; d) Methyl (triphenylphosphoranylidene)​acetate, DCM; e) 10% Pd/C, H_2_, CH_3_COOH, THF; f) LiAlH_4_, THF.

**Scheme S2**

^a^**Reagents and conditions:** a) 10% Pd/C, H_2_, THF; b) LiAlH_4_, THF.

**Scheme S3**

**^a^Reagents and conditions**: a) NaBH_4_, THF; b) PPh_3_CH_3_I, NaH, DMF; c) BH_3_, THF, H_2_O_2_, NaOH.

**Scheme S4**

**^a^Reagents and conditions**: a) Acetyl chloride, TEA, DCM

**Scheme S5**

**^a^Reagents and conditions**: a) DIBAL-H, THF.

**Scheme S6**

**^a^Reagents and conditions**: a) (Carbethoxymethylene)triphenylphosphorane, DCM; b) Triethyl 2-fluoro-2-phosphonoacetate, DBU, LiCl, THF; c) DICAL-H, THF.

**Scheme S7**

^a^**Reagents and conditions:** a) 10% Pd/C, PPh_3_, CuI, Et_3_N, H_2_O; b) Methyl (triphenylphosphoranylidene)​acetate, CH_2_Cl_2_; c) 10% Pd/C, H_2_, CH_3_COOH, THF; d) LiAlH_4_, THF.


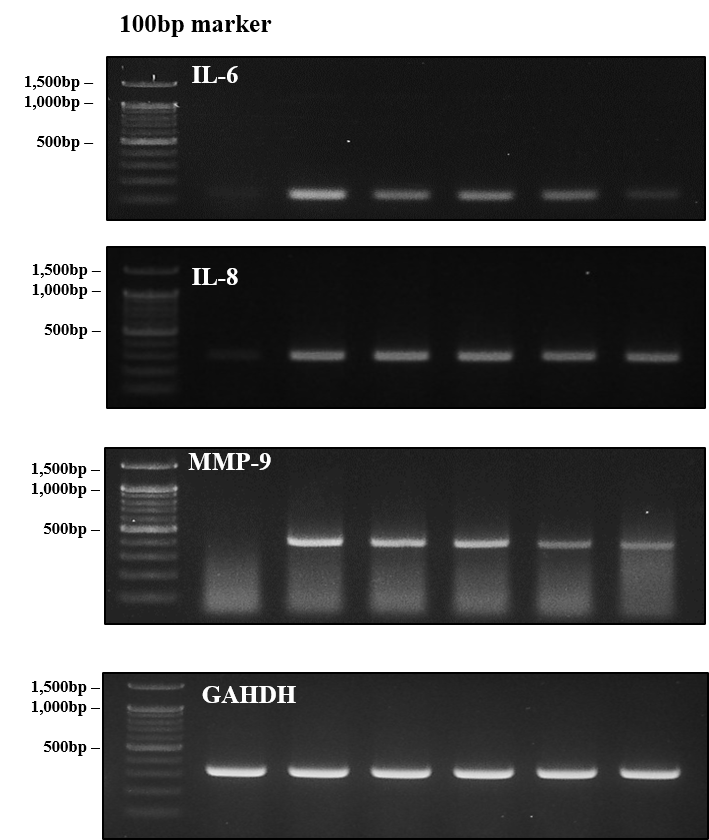


**Fig S1. Original PCR for image used in Fig 1e.**

Each PCR product was a clear band and detected at the expected size.

For detection of PCR product and GAPDH, relevant portions of the gel were scanned using the imaging system.


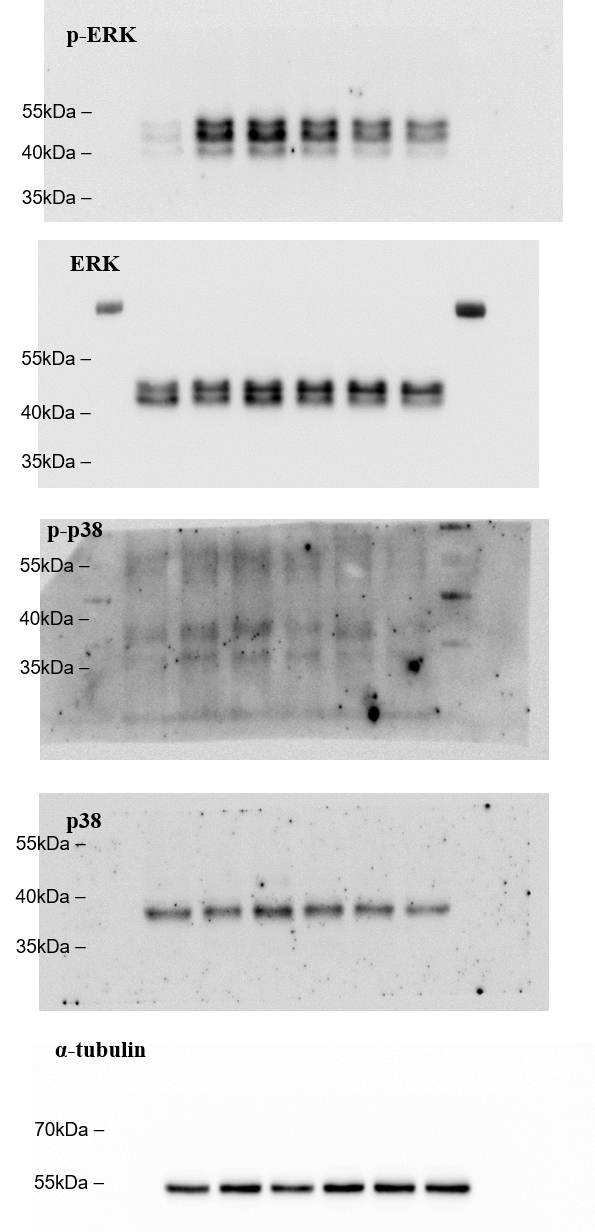


**Fig S2. Original western blots for image used in Fig 2a.**

Each antibody produced a clear band at the expected size.

For detection of phosphor and total proteins, the imaging system was used and relevant portions of the blot were scanned


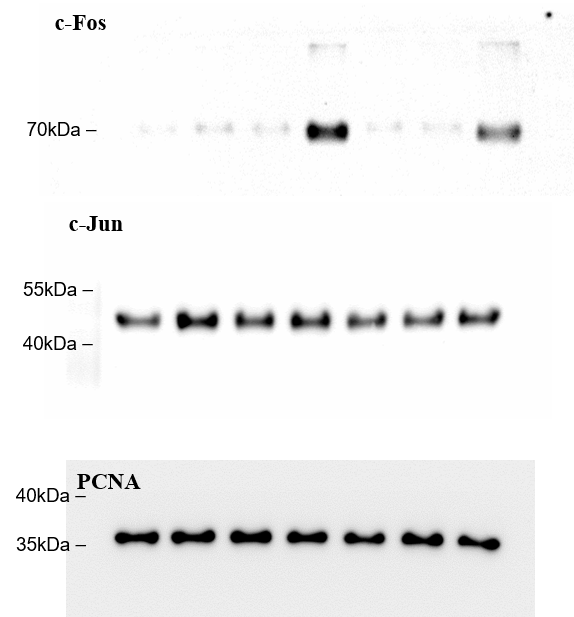


**Fig S3. Original western blots for image used in Fig 2c.**

Nuclear extracts were isolated from the cells using a Pierce kit and proteins analyzed by western blot. Each antibody produced a clear band at the expected size.

For detection of proteins and PCNA, the imaging system was used and relevant portions of the blot were scanned


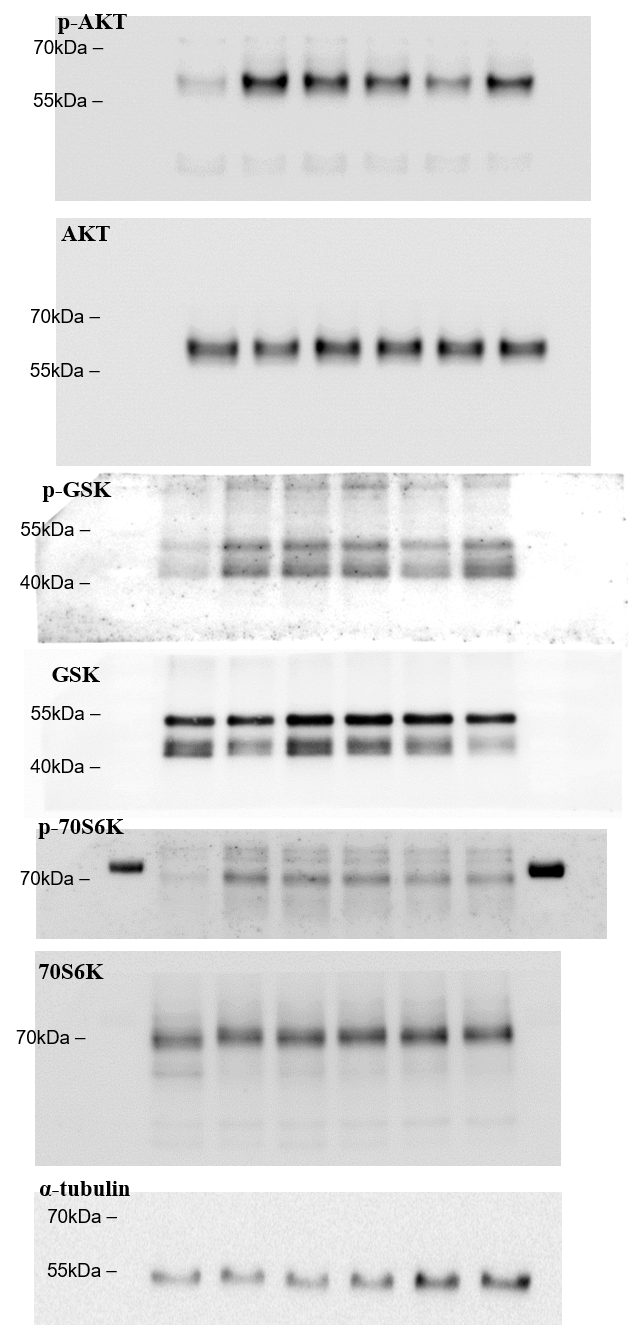


**Fig S4. Original western blots for image used in Fig 3a.**

Each antibody produced a clear band at the expected size.

For detection of phospho and total proteins, standard, the imaging system was used and relevant portions of the blot were scanned
